# Regulation of the Intestinal Extra-Adrenal Steroidogenic Pathway Component LRH-1 by Glucocorticoids in Ulcerative Colitis

**DOI:** 10.3390/cells11121905

**Published:** 2022-06-12

**Authors:** Glauben Landskron, Karen Dubois-Camacho, Octavio Orellana-Serradell, Marjorie De la Fuente, Daniela Parada-Venegas, Mirit Bitrán, David Diaz-Jimenez, Shuang Tang, John A. Cidlowski, Xiaoling Li, Hector Molina, Carlos M. Gonzalez, Daniela Simian, Jaime Lubascher, Victor Pola, Martín Montecino, Tjasso Blokzijl, Klaas Nico Faber, María-Julieta González, Rodrigo Quera, Marcela A. Hermoso

**Affiliations:** 1 Innate Immunity Laboratory, Immunology Program, Biomedical Sciences Institute, Faculty of Medicine, Universidad de Chile, Santiago 8380453, Chile; glandskron@uft.cl (G.L.); kdubois@med.uchile.cl (K.D.-C.); octavio.orellana.s@gmail.com (O.O.-S.); mdelafuente@uft.cl (M.D.l.F.); dparadav@gmail.com (D.P.-V.); mirit.bitran@ug.uchile.cl (M.B.); hectormol@gmail.com (H.M.); 2 Biomedicine Research Laboratory, Medical School, Universidad Finis Terrae, Santiago 7501015, Chile; 3 Department of Gastroenterology and Hepatology, University Medical Center Groningen, University of Groningen, 9713 GZ Groningen, The Netherlands; t.blokzijl@umcg.nl (T.B.); k.n.faber@umcg.nl (K.N.F.); 4 Molecular Endocrinology Group, Signal Transduction Laboratory, National Institute of Environmental Health Sciences, National Institutes of Health, Department of Health and Human Services, Research Triangle Park, Durham, NC 27709, USA; david.diaz-jimenez@nih.gov (D.D.-J.); cidlows1@niehs.nih.gov (J.A.C.); 5 Metabolism and Nuclear Medicine Group, Fudan University Cancer Institute, Fudan University Shanghai Cancer Center, Shanghai 200433, China; tangshuang@fudan.edu.cn; 6 Metabolism, Genes, and Environment Group, Signal Transduction Laboratory, National Institute of Environmental Health Sciences, National Institutes of Health, Department of Health and Human Services, Research Triangle Park, Durham, NC 27709, USA; lix3@niehs.nih.gov; 7 School of Veterinary Medicine, Faculty of Life Sciences, Universidad Andrés Bello, Santiago 8370251, Chile; carlosgonzalez@unab.cl; 8 Academic Research Unit, Clínica Las Condes, Santiago 7591018, Chile; danielasimian@gmail.com; 9 Inflammatory Bowel Disease Program, Gastroenterology Department, Clínica Las Condes, Santiago 7591018, Chile; jlubascher@clinicalascondes.cl; 10 Institute of Biomedical Sciences, Faculty of Medicine and Faculty of Life Sciences, Universidad Andrés Bello, Santiago 8370186, Chile; vpola02@ug.uchile.cl (V.P.); mmontecino@unab.cl (M.M.); 11 FONDAP Center for Genome Regulation, Santiago 8370146, Chile; 12 Cell and Molecular Biology Program, Biomedical Sciences Institute, Faculty of Medicine, Universidad de Chile, Santiago 8380453, Chile; jgonzale@med.uchile.cl; 13 Centro de Enfermedades Digestivas, Programa Enfermedad Inflamatoria Intestinal, Clínica Universidad de Los Andes, Universidad de los Andes, Santiago 7620157, Chile; rquera@clinicauandes.cl

**Keywords:** LRH-1, glucocorticoid receptor, ulcerative colitis, steroid refractoriness, steroid dependency

## Abstract

Ulcerative colitis (UC) is an inflammatory bowel disease (IBD) and can be treated with glucocorticoids (GC), although some patients are unresponsive to this therapy. The transcription factor LRH-1/*NR5A2* is critical to intestinal cortisol production (intestinal steroidogenesis), being reduced in UC patients. However, the relationship between LRH-1 expression and distribution with altered corticosteroid responses is unknown. To address this, we categorized UC patients by their steroid response. Here, we found that steroid-dependent and refractory patients presented reduced glucocorticoid receptor (GR)-mediated intestinal steroidogenesis compared to healthy individuals and responder patients, possibly related to increased colonic mucosa GR isoform beta (GRβ) content and cytoplasmic LRH-1 levels in epithelial and lamina propria cells. Interestingly, an intestinal epithelium-specific GR-induced knockout (GR^iKO^) dextran sodium sulfate (DSS)-colitis mice model presented decreased epithelial LRH-1 expression, whilst it increased in the lamina propria compared to DSS-treated control mice. Mechanistically, GR directly induced *NR5A2* gene expression in CCD841CoN cells and human colonic organoids. Furthermore, GR bound to two glucocorticoid-response elements within the *NR5A2* promoter in dexamethasone-stimulated CCD841CoN cells. We conclude that GR contributes to intestinal steroidogenesis by inducing LRH-1 in epithelial cells, suggesting LRH-1 as a potential marker for glucocorticoid-impaired response in UC. However, further studies with a larger patient cohort will be necessary to confirm role of LRH-1 as a therapeutic biomarker.

## 1. Introduction

Glucocorticoids (GC) are the traditional front-line therapy for moderate or severe ulcerative colitis (UC) [1]. However, 60% of IBD patients treated with GC achieve clinical and endoscopic remission, with 20% becoming steroid-dependent to maintain remission, and a further 16–20% being steroid-refractory [2,3]. A refractory response to GC in IBD patients has been associated with glucocorticoid receptor β isoform (GRβ) protein content and single nucleotide polymorphisms (SNPs) present in the *NR3C1* gene (encoding GR) [4,5], indicating a GR functional role in steroid response failure.

Endogenous cortisol production is classically associated with the adrenal glands and gonads, but other organs, such the brain, lung, thymus, skin, liver, and intestine also produce corticoids [6]. A GR role in activating adrenal steroidogenesis has been demonstrated by upregulated expression of the cytochrome P450 enzymes A1 and B1 (*CYP11A1* and *CYP11B1*) [7], mainly controlled by the transcription factor SF-1 [8]. CYP11A1 is involved in the first step of cortisol synthesis by cholesterol conversion into pregnenolone (a precursor of most steroid hormones) and subsequent catalysis of 11-deoxycortisol to cortisol by CYP11B1 [9]. Although extra-adrenal steroidogenesis was seen in small and large intestinal crypt epithelial cells (perhaps regulating local immune response) [10], the pathways involved in gut steroidogenesis are not fully understood.

A key step in intestinal steroidogenesis is the transcription regulator LRH-1 (encoded by the *NR5A2* gene), which is homologous to the adrenal steroidogenic factor SF-1 (encoded by *NR5A1*) mainly expressed in gut epithelial cells, impacting proliferation and cell renewal [11,12]. Additionally, intestinal mucosa and epithelial cell lines exposed to inflammatory stimuli, such as phorbol ester (PMA) or lipopolysaccharide (LPS), secrete cortisol through a pathway involving LRH-1 [13,14]. T-cells also express LRH-1 and steroidogenic enzymes, suggesting they also have the potential to produce cortisol [15,16]. Furthermore, reduced LRH-1 expression was observed in the inflamed mucosa of IBD patients [17,18], and mice lacking enterocyte-specific LRH-1 were found to be more susceptible to dextran sodium sulfate (DSS)-induced colitis, suggesting that intestinal steroidogenesis regulates mucosal inflammation [17].

For the most part though, regulation of intestinal steroidogenesis and its impact on steroid responsiveness in IBD patients is still not fully understood. Moreover, microarray analysis data for inflammatory human macrophages showed a two-fold increase in *NR5A2* gene expression and a three-fold increase when cells were treated with Dex (a synthetic GC), compared to untreated cells [19], strongly suggesting that GR is a molecular regulator of LRH-1 transcription.

In this study, we explore the steroidogenic LRH-1 transcription factor expression pattern in the intestinal mucosa of UC patients and healthy subjects, with special emphasis on its relationship to patient steroid response, and in an intestinal-epithelium-specific GRiKO colitis mice model. Additionally, we investigate the GR-mediated mechanism of cortisol production involving the molecular regulation of the LRH-1 gene.

## 2. Materials and Methods

### 2.1. Human Samples

This prospective observational study included colonoscopy samples from ulcerative colitis (UC) adult patients and healthy individuals of both genders obtained at the Gastroenterology Department of Clínica Las Condes (Santiago, Chile) between February 2018 and January 2020. The study data were collected and managed using REDCap electronic data capture tools hosted at Clínica Las Condes. A diagnosis of UC was established according to international guidelines including clinical, endoscopic and histologic criteria. Patients undergoing colonoscopy with an endoscopic Mayo score > 2, were invited to participate in this study, and were required to start treatment with GC (0.75 mg/kg/day of prednisolone, 1 mg/kg/day of prednisone or an equivalent treatment) [20] immediately after the colonoscopy procedure. Healthy individuals were subjects who underwent colonoscopy control without any of the exclusion criteria: IBD, autoimmune disease, severe food allergies, celiac disease, diverticulitis, irritable bowel syndrome or any concurrent infection.

Colonoscopy pinch mucosa biopsies (six biopsies per subject, taken from zones close to each other of 2–3 mm diameter each with the forceps model EndoJaw FB-230U, Olympus ^®^Brooklyn Park, MN, USA) of the most inflamed area in UC patients and from the sigmoid in healthy individuals, were fixed using 2% paraformaldehyde, then paraffin-embedded, or stored in RNA later for further IHC and RT-qPCR analysis, respectively. Fresh samples were placed in DMEM/F12 medium (P/S 1%; gentamicin and fungizone) for ex vivo stimulus to measure cortisol and cytokine production.

Patients were grouped according to GC response as: (1) responsive (GC-R)—patients that achieved clinical response within two weeks with full GCs treatment and remained in clinical remission after three months of stopping GC treatment; (2) steroid-dependent (GC-D)—patients with an inability to handle systemic steroids below 10 mg prednisolone within three months without recurrent active disease, or symptomatic relapse of IBD within three months of stopping steroids; and (3) steroid-refractory (GC-Rf)—patients with an inability to experience symptomatic remission with systemic steroids (prednisolone up to 0.75 mg/kg/day over a period of four weeks) [21].

All participants provided informed consent. The study was approved by the Medical Direction and Local Ethics Committee of Clinica Las Condes (approved 8 August 2014, reference number J032017) and was performed according to human experimental guidelines. Clinical investigations were conducted according to the Declaration of Helsinki principles with participants identified only by number.

### 2.2. Reagents

Detailed reagent information is included in the Supplementary Materials.

### 2.3. Ex Vivo Culture of Intestinal Biopsies

Intestinal biopsies were transported in DMEM/F12 medium 0.1 mg/mL gentamicin, 0.5 µg/mL fungizone and 0.5 mg/mL normocin until processing. Biopsies were washed twice with PBS and placed in 24-well plates with 350 µL of DMEM/F12 medium plus antibiotics, and treated with 100 nM Dex, 10 µM RU-486, 20 µM SR-1848, Dex plus RU-486, Dex plus SR-1848 and DMSO as control for 24 hours (h). Afterwards, media were collected and centrifuged for debris clearance (1200 rpm per 5 min). The supernatants were frozen for further cortisol and CBA assays.

### 2.4. Human Colonic Organoids

Colon tissue was taken from the healthy flanking regions in surgical resection specimens of colon cancer patients, after obtaining informed consent. Crypt-derived colonic organoids were generated and cultured as described by Sato et al. [22,23], using an expansion medium with human recombinant proteins (Rspo-1, noggin, B27, NAC, IGF1, FGF-2, EGF, nicotinamide, SB202190, A83) and Wnt3-conditionated medium [24]. Organoids were grown in Cultrex Ultimatrix (R&D Systems, Minneapolis, MN, USA) and cultured in expansion medium for 72 h, followed by exposure for 24 h to 100 nM dexamethasone. Next, organoids were harvested for RT-qPCR and immunofluorescence microscopy (IF) analysis.

### 2.5. Cell Line In Vitro Experiments

The human colon cell line CCD841CoN was obtained from the American Type Culture Collection (Rockville, MD, USA, catalog number CRL-1790) and cultured in 10% FBS-supplemented MEM with 1% P/S at 37 °C in 5% CO_2_ in a humid environment. For in vitro experiments, 1 × 10^6^ cells/mL were seeded in 6-well plates. After complete cell attachment, cells were washed with PBS and the medium replaced by MEM supplemented with 1% charcoal stripped FBS. Prior to dexamethasone treatment, cells were treated with RU-486 (10 µM) or SR-1848 (20 µM) for 1 h Dexamethasone treatment was conducted for 24 h prior to harvesting cells and supernatant for further cortisol and gene expression evaluation.

### 2.6. RT-qPCR Assays

The mRNA expression analyses were performed with 100 ng of cDNA for each assay using either TaqMan-specific expression probes (Thermo Fisher Scientific, Carlsbad, CA, USA. Cat. 4331182) for *NR5A2*, *NR3C1* and *NR3C1* isoform β, *TSC22D3*; mouse *Nr5a2/Lrh-1* or the Brilliant^®^ II kit SYBR^®^ Green QPCR Master Mix (Agilent Technologies Inc., Santa Clara, CA, USA) for *CYP11A1* and *CYP11B1*. qPCR was performed in an Mx3000 P QPCR System (Agilent Technologies Inc., Santa Clara, CA, USA). To analyze the qPCR results, the ∆∆C_q_ method was used; *PPIB* or *18S* RNA (*Ppib* or *18s* for mouse) were used as housekeeping genes after validation assays.

### 2.7. Immunohistochemistry (IHC)

The IHC procedure and analysis are detailed in the Supplementary Materials.

### 2.8. Animal Experimentation

Intestinal-epithelium-specific GR knockout mice (GR^iKO^; Villin^−^cre^+^, *Nr3c1*^flox/flox^) and their littermate control GR^flox^ (Villin^−^cre^−^, *Nr3c1*^flox/flox^) on the C57BL/6J background were generated by crossing mice carrying the floxed GR allele (*Nr3c1*^loxP/loxP^) [25,26] with Villin^−^cre mice (#004586, Jackson Laboratory Ben Harbor, ME) [27]. GR^iKO^ and GR^flox^ mice aged 6 months were housed in the National Institute of Environmental Health Sciences (NIEHS) animal facility rooms and maintained under strict specific pathogen-free conditions. Age- and gender-matched mice were used for all experiments.

Acute colitis was induced by adding 2.5% DSS in the drinking water for 7 consecutive days. GR^flox^ (*n* = 10) and GR^iKO^ (*n* = 8) DSS-treated mice were monitored for body weight, rectal bleeding, stool consistency and survival, with a disease activity index (DAI) calculated according to Cooper et al. (total score [body weight decrease + stool consistency + rectal bleeding]/3) [28].

After the treatment, the mice were euthanized, and colons were excised upon autopsy and colon lengths were measured. The colonic tissues were further fixated in 10% formalin for histopathology studies, and histological analysis from Erben and colleagues [29] was performed. The total histological score included variables related to the quality and dimensions of the inflammatory cell infiltrates, epithelial changes, and mucosal architecture. The animal protocol was approved by the ethics committees of the Faculty of Medicine at the Universidad de Chile and the National Institute of Environmental Health Sciences (NIEHS) (CBA #0953 FMUCH; ASP 2014-0016 LST, NIEHS).

### 2.9. Immunofluorescence of Slides from Patient’ Biopsies, Murine DSS-Induced Models and Organoid Experiments

Indirect immunofluorescence of colon mucosa from patient groups (according to GR response), GR^flox^ (WT) and GR^iKO^ +/− DSS mice models, and organoids stimulated +/− Dex, was performed on paraffin-embedded samples evaluating LRH-1 and GR co-expression with E-cadherin. Blank slides from mice models were evaluated for CYP11A1 expression. The slides were subjected to deparaffinization (NeoClear, Merck KGaA, Darnstadt, Germany) and then rehydrated with different ethanol concentrations. Primary antibodies were the same as were used in IHC, plus anti-E-cadherin for epithelium co-localization, followed by incubation with conjugated secondary antibodies (Thermo Scientific, Waltham, MA, USA). Hoechst 33,342 (Thermo Scientific, Waltham, MA, USA) was used as a nuclear counterstain. Finally, slides were covered with a coverslip plus mounting solution (Dako, Agilent Technologies Inc., Santa Clara, CA, USA) and visualized under a Nikon C2+ confocal microscope at 20X objective (Nikon Instruments Inc., Melville, NY, USA). Image compositions were carried out using FIJI/Image J Software (Version 1.53q), subtracting background (25 pixels) to all channels to reduce the signal-to-noise ratio. Percentage quantification of positive and negative nuclei relative to total for LRH-1 and GR proteins in organoid IF was carried out with FIJI/Image J Software segmenting the nuclear region of interest from four randomly chosen organoids.

### 2.10. Transcriptome Array

Gene expression microarrays from colon samples from C57BL/6 mice GR^flox^ and GR^iKO^ +/− DSS mouse models (GR^flox^Ctol *n* = 4; GR^iKO^ Ctol *n* = 4; GR^flox^ DSS *n* = 3; GR^iKO^ DSS *n* = 3) were conducted using Agilent whole mouse genome 4 × 44 multiplex format oligo arrays (014868) (Agilent Technologies) following the Agilent 1-color microarray-based gene expression analysis protocol. Starting with 500 ng of total RNA, Cy3-labeled cRNA was produced according to the manufacturer’s protocol. For each sample, 1.65 mg of Cy3-labeled cRNA was fragmented and hybridized for 17 h in a rotating hybridization oven. Slides were washed and then scanned with an Agilent scanner. Data was obtained using the Agilent feature extraction Software (Version 12.0), using the 1-color defaults for all parameters. The Agilent feature extraction software performed error modeling, adjusting for additive and multiplicative noise. The resulting data were processed using Omicsoft Array Studio Software (Version 7.0). The Gene Expression Omnibus accession number of this dataset is GSE146048 (https://www.ncbi.nlm.nih.gov/geo/query/acc.cgi?acc=GSE146048), last accessed on 20 December 2021.

### 2.11. Cortisol Measurement

Cortisol concentrations were measured in supernatants of human colonic biopsies cultured in 350 ul with different treatments (Dex, RU-486, SR-1848) using the ELISA kit DetectX Cortisol Enzyme Immunoassay Kit (Arbor Assays, Ann Arbor, MI, USA, Cat. K003-H5), as detailed in the Supplementary Materials. Ex vivo biopsies and cell line supernatant samples were diluted 1:10. Considering the possible cross-reactivity of Dex in cortisol detection, we prepared an internal control of a medium containing 100 nM Dex (‘Dex-control’) diluted 1:10. The concentration obtained in this internal control was subtracted from each Dex-stimulated sample (see formula below). The concentration range for the Dex-control was between 0.1–0.20 ng/mL. Dex-treated sample concentrations, minus internal control and dilution factor application ranged between 2.0–10.0 ng/mL. The standard curve concentration ranged between 0.05–3.2 ng/mL. The Dex internal control concentration was less than 10% of the Dex-treated experimental samples. Data analysis was performed using a 4PLC fitting curve and interpolated from the standard curve.

Cortisol content = 100 nM Dex-treated samples (1:10) − Dex-control (100 nM Dex-treated culture medium diluted 1:10) × dilution factor (10)

### 2.12. Cytometric Bead Array (CBA)

Cytokine secretion (TNF, IFN-γ, IL-2, IL-6, IL-4, IL-10 and IL-17A) by ex vivo explants culture was detected using a BD^TM^ CBA Human T_H1_/T_H2_/T_H17_ Kit (BD Biosciences, San Jose, CA, USA) following the manufacturer’s instructions. Briefly, a supernatant of mucosa culture exposed to different treatments (Dex, RU-486 or SR-1848) was incubated with a mixture of anti-cytokine capture antibodies-conjugated beads and PE-conjugated detector antibodies for 3 h at room temperature in the dark, subsequently washed with 1× wash buffer and centrifuged at 200× *g* for 5 min at room temperature. Data was acquired using a FACS CantoTM II flow cytometer (BD) and analyzed using BD Cytometric Bead Array software (Version 1.4) (BD).

### 2.13. In Silico Analysis of GREs in Human NR5A2 Gene, Immunoprecipitation of Chromatin Coupled to Real Time PCR and Formaldehyde-Assisted Isolation of Regulatory Elements (FAIRE) Analysis

Analysis in silico using the JASPAR database [30] revealed the presence of putative GREs in the *NR5A2* promoter region. These GREs were mapped and analyzed by multiple alignments against the consensus sequence using the STAMP software, demonstrating the likelihood of GR binding in strand (+) of the DNA promoter. According to this, primers flanking each GRE in the *NR5A2* promoter region were used for ChIP-qPCR. For this experiment, 9 × 10^6^ of CCD841CoN cells were cultured as previously described and treated with Dex for 2 h. Cells were then fixed and cross-linked using PFA 1% for 10 min at room temperature, followed by reaction-quenching with 1× glycine for 5 min, and immunoprecipitated using the EZ-Magna ChIP™ A/G kit (Merck Millipore, Burlington, MA, USA). The ChIP assay is detailed in the Supplementary Materials.

### 2.14. Western Blotting

Proteins from experiments with the CCD841CoN cells were extracted using a RIPA Lysis Buffer (Thermo Scientific, Carlsbad, CA, USA), and quantified using a Pierce™ BCA Protein Assay kit (Thermo Scientific). The following antibodies were used: LRH-1 (Novus-Bio, Centennial, CO, USA, Cat. NBP1-32489) and β-actin as load control (Santa Cruz Biotechnology Dallas, TX, USA, Cat. Sc-4778). A recombinant LRH-1 protein was used as a positive control (Novus-Bio, H00002494-P01).

### 2.15. Statistical Analysis

First, the normal distribution of data was evaluated using the D’Agostino and Pearson normality test. Results with a normal distribution were expressed as the mean plus standard error, using an unpaired *t*-test or one-way ANOVA with Bonferroni or Tukey post-tests for comparison of quantitative variables. Non-parametric data were expressed as the median plus interquartile range and Mann–Whitney or Kruskal–Wallis tests with Dunn’s post-test were used for comparison of different variables. A Friedman post-test was used for comparison of paired analysis. The percentage quantification of positive and negative nuclei relative to the total for LRH-1 and GR proteins in dexamethasone-treated organoids versus control was analyzed using a chi-squared test. Statistical analysis was performed with GraphPad Software 8.0 considering a significant *p*-value less than 0.05. Microarray analysis for differentially expressed genes was determined using ANOVA with cutoff (FDR adjusted *p*-value (*q*-value) < 0.05) using the Partek Genomics Suite. Functional enrichment of significantly differentiated expressed genes were analyzed by Ingenuity Pathway Analysis software (Version 8.7) (IPA, Ingenuity systems Inc, Redwood City, CA, USA).

## 3. Results

### 3.1. Dexamethasone Regulates Steroidogenesis in Colonic Mucosa from Steroid-Responder UC Patients

As previously shown, GC may induce steroidogenesis in adrenal gland cells by upregulation of CYP11A1 and CYP11B1 expression [7]. As GR expression is associated with impaired GC treatment responses in UC [31], we determined if GC treatment induces cortisol production in intestinal mucosa. This analysis included 10 healthy individuals (H) and 21 patients showing active UC—13 responders to steroid therapy (UC-R), 4 dependent (UC-D) and 4 refractory patients (UC-Rf). The clinical characteristics of each group are shown in Table 1.

**Figure 1:**
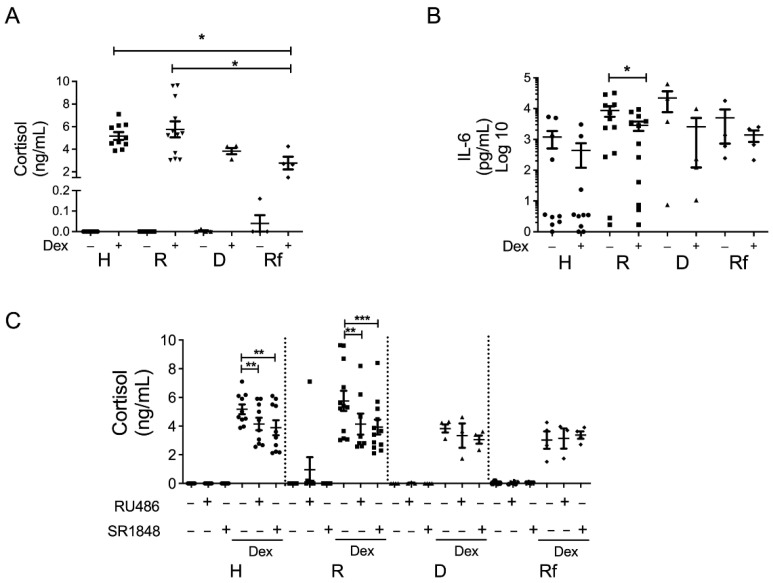
GR-dependent cortisol production in intestinal mucosa. (**A**) Cortisol content in supernatant of biopsies from healthy controls (H), responders (R), dependent (D) and refractory (Rf) UC patients, stimulated with or without 100 nM Dex and/or inhibitors for 24 h; (**B**) IL-6 levels after Dex treatment; and (**C**) cortisol levels after treatment with Dex and GR (RU-486) or LRH-1 (SR-1848) inhibitors. Differences between medians were assessed using Kruskal–Wallis test (**A**), Friedman test (**B**) and (**C**) with Dunn’s post-test, * *p* < 0.05; ** *p* < 0.01, *** *p* < 0.001. Each point represents an individual value: healthy (circles), responders (squares), dependents (triangles) and refractory (diamonds).

Therefore, using a gut explant model, inflamed colonic biopsies from UC patients and non-inflamed biopsies from healthy individuals were cultured ex vivo for 24 h, with dexamethasone (100 nM), and/or pretreated with RU-486 or SR-1848 (GR and LRH-1 antagonists) determining the role of GR and LRH-1 in cortisol production and cytokine secretion in supernatants. Cortisol production was undetectable in the non-stimulated condition for the majority of ex vivo mucosa samples as shown previously [13]. Nevertheless, dexamethasone induced cortisol production in all patient groups (Figure 1A). Interestingly, cortisol production was lower in UC-Rf compared to the control and UC-R patients (*p* = 0.008 and 0.013, respectively). There was no significant difference between the UC-Rf and UC-D patient groups (*p* = 0.69).

The cytokine levels in the supernatants demonstrated that TNF, IL-6 and IL-10 were higher in the UC-R group compared to the control (*p* = 0.003, 0.036, 0.007, respectively), and IL-4, IL-10 and IL-17A were higher in the UC-D group compared to the control (Supplementary Figure S1), *p* = 0.028, 0.007 and 0.039, respectively). In addition, UC-Rf mucosa secreted higher TNF and IL-10 levels than control mucosa (*p* = 0.013 and 0.007), indicating that a complex inflammatory profile was represented in the UC patient samples (Supplementary Figure S1). Moreover, upon Dex treatment, lower IL-6 levels were observed in UC-R patient mucosa (Figure 1B, *p* = 0.042), as previously shown [32]; other cytokines showed similar levels after Dex-treatment.

Additionally, we analyzed the GR and LRH-1 role in Dex-induced cortisol production using RU-486 or SR-1848 in explant cultures, respectively, finding cortisol levels partially reversed with both inhibitors in healthy (*p* = 0.002 for both, RU-486 and SR-1848) and UC-R patient samples (*p* = 0.054 and 0.001, respectively). However, UC-D and UC-Rf patients showed no inhibition by both compounds (Figure 1C).

These results demonstrate that the UC-D and UC-Rf patient intestinal mucosa exhibited impaired GR functions affecting the steroidogenesis pathway in cortisol production.

### 3.2. Steroidogenesis Pathway Components in Intestinal Mucosa of UC Patients

**Figure 2:**
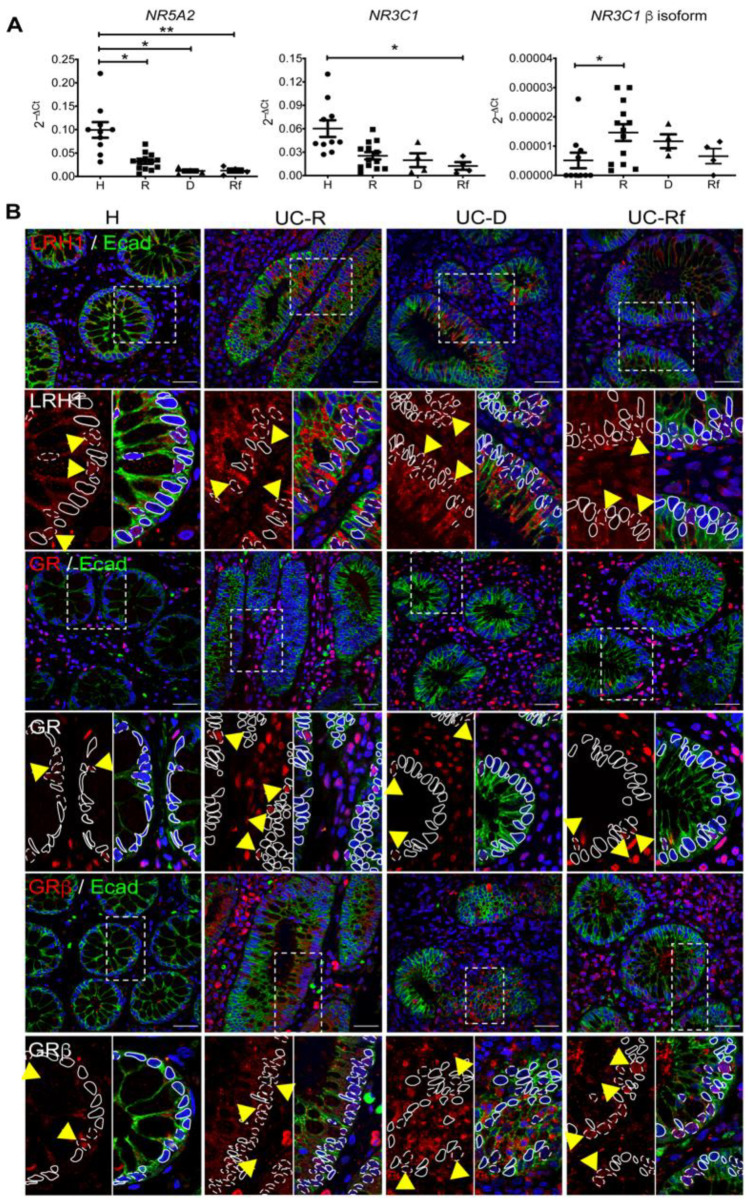
LRH-1 and GRβ are upregulated in intestinal mucosa from patients with impaired GC response. (**A**) Transcript levels (relative to *PPIB*) of *NR5A2, NR3C1* and *NR3C1* isoform β in intestinal mucosa from healthy controls (H), responders (R), dependent (D) and refractory (Rf) UC patients by RTqPCR. Transcript levels (relative to *PPIB*) of *NR5A2*, *NR3C1* and *NR3C1* isoform β; differences between group medians were assessed using Kruskal–Wallis with Dunn’s post-test and Pearson correlation performed. Each point represents an individual value: non-inflamed (circles), responders (squares), dependents (triangles) and refractory (diamonds), * *p* < 0.05; ** *p* < 0.01. (**B**) Representative immunoreactivity of LRH-1, GR and GRβ isoform (red) in paraffin-embedded PFA-fixed sections of intestinal mucosa biopsies taken from healthy individuals and active UC patients according to their GC response by tissue array. E-cadherin (Ecad) co-localization (green) was used for epithelium recognition. In the zoomed image indicated with dashed line and yellow arrow: positive nuclear stain; solid line: negative nuclear stain. Objective 60×, scale bar 30 μm.

As GR-mediated cortisol production in intestinal mucosa was reduced in UC-Rf (Figure 1A), we then determined basal transcript levels of the intestinal steroidogenic pathway main components (*NR5A2*, *CYP11A1*), together with the *NR3C1* and *NR3C1* β isoforms in intestinal mucosa biopsies. The steroidogenic transcription factor *NR5A2* mRNA was reduced in all UC-patients compared to healthy controls, with no difference between each UC group (Figure 2A, *p* = 0.0251, 0.0154 and 0.0041 for R, D and Rf, respectively vs. H). *NR3C1* transcript levels were decreased in UC-Rf compared to controls (*p* = 0.0126), whereas the *NR3C1* isoform β was increased in UC-R compared to controls (Figure 2A, *p* = 0.0463). Transcripts for the steroidogenic enzyme *CYP11A1* showed no differences amongst groups (Supplementary Figure S2), and transcripts for *CYP11B1* were below the detection limit (Ct > 38 cycles) in all samples (using an in-house design primer pair or a commercial TaqMan probe). As *NR5A2*, *CYP11A1* and *NR3C1* mRNA levels were measured in bulk tissue, we next evaluated protein content and distribution by IHC in a tissue microarray (TMA). LRH-1 showed nuclear and cytoplasmic staining in epithelial and lamina propria cells (Supplementary Figure S3A), with similar epithelial immunoreactivity in all UC groups and healthy controls, and increased staining in the lamina propria of UC-R and UC-D patients, compared to controls (*p* = 0.0376 and 0.0191, respectively) (Supplementary Figure S3B). Additionally, confocal analysis demonstrated a strong LRH-1 staining in the cytoplasm of epithelial and LP immune cells from UC patients. Interestingly, nuclear LRH-1 staining was mainly present in epithelial cells from controls, whereas the UC patients exhibited a lower proportion of positive nuclei in epithelial cells (Figure 2B).

Immunoreactivity of GR and GRβ was predominant in both epithelium and lamina propria cells (Supplementary Figure S3A). Quantification of GR showed decreased epithelial expression in UC-R and UC-D patients compared to controls (Supplementary Figure S3B, *p* = 0.0006 and 0.014, respectively). Meanwhile, GRβ positivity was increased in crypt epithelial cells (Supplementary Figure S3B, *p* = 0.014 and 0.04, respectively) and lamina propria (*p* = 0.005 and 0.01, respectively) from UC-D and UC-Rf compared to healthy controls; interestingly, GRβ staining showed an increased immunoreactivity in UC-D vs. UC-R in the epithelium (*p* = 0.02). CYP11A1 protein expression showed no difference in both epithelium and LP cells among groups (Supplementary Figure S3A,B).

These data showing LRH-1 cytoplasmic localization and increased GRβ content (epithelial and infiltrate compartments) in UC-D and UC-Rf partly explain the impaired cortisol production by mucosal tissue exposed to Dex (Figure 1A).

### 3.3. GRiKO DSS Mouse Model Shows Strong Upregulation of Inflammatory Mediators and Spatially Distinct LRH-1 Expression in Intestinal Mucosa

To confirm observations of the relationship between intestinal steroidogenesis and GR signaling regarding local inflammation, we developed a DSS-colitis intestinal epithelium-specific GR KO (GR^iKO^) mice model. Deletion of GR exacerbated DSS-induced mice colitis with decreased colon length, increased erosion, rectal bleeding, tissue damage and enhanced intestinal inflammation, as seen by hematoxylin/eosin staining here and as previously reported [33] (Supplementary Figure S4, Table 2).

**Figure 3:**
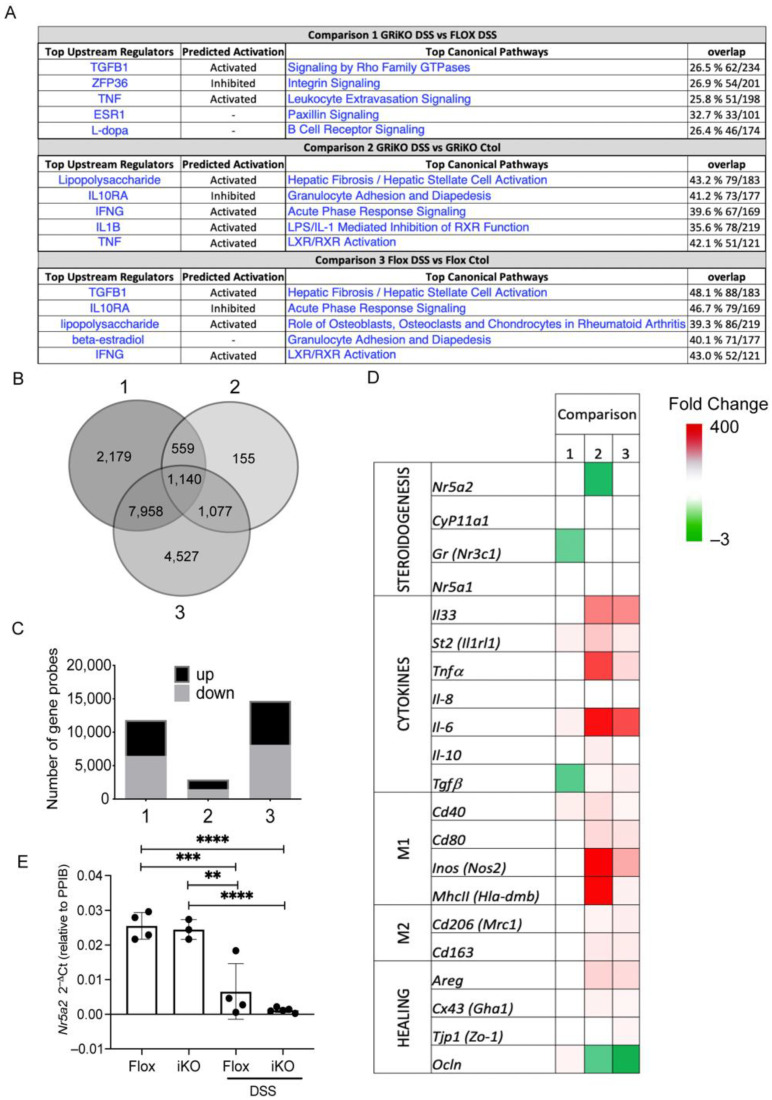
Epithelial GR modulates gene expression in intestinal mucosa from DSS-treated mice. (**A**) Top five upstream regulators and canonical pathways from gene expression microarray in GR^iKO^ DSS vs. GR^flox^ DSS (Comparison 1), GR^iKO^ DSS vs. GR^iKO^ Ctol (Comparison 2), and GR^flox^ DSS vs. GR^flox^ Ctol (Comparison 3) using Ingenuity Pathway Software (IPA); (**B**) Venn diagram of the overlap in significant gene probes determined from 3 comparisons; (**C**) Number of gene probes either induced (**up**) or repressed (**down**) by each comparison; (**D**) Gene probes that showed statistically significant differences for any of the three comparisons and that affected immune response and steroidogenesis pathway were selected and grouped into five categories: (1) steroidogenesis, (2) cytokines, (3) M1-like markers, (4) M2-like markers and (5) re-epithelialization. The heatmap indicates increased (red/upregulated) or decreased (green/downregulated) expression levels. Two-way ANOVA (*p* < 0.05); (**E**) *Nr5a2* transcript (relative to *Ppib*) from colonic samples from each mice group. one-way ANOVA with Tukey’s post-test.** *p* < 0.01, *** *p* < 0.001, **** *p* < 0.0001.

To have an overview of the genes and signaling pathways affected by the presence/absence of GR and inflammation, we used microarray assays in the GR^iKO^ mice model (Figure 3). We determined the differential gene expression in inflamed (DSS treated)/non-inflamed (vehicle treated) colon samples from GR^iKO^ or control (GR^flox^) mice using the IPA software (Figure 3A). Comparisons between GR^iKO^ DSS and GR^flox^ DSS (Comparison 1) mice showed that TGFB1, and TNF were the upstream regulators predicted as active, and ZFP36 (a GC gene target [34]) was inhibited, while signaling by Rho GTPases, integrin signaling, and leukocyte extravasation were the most commonly differentially affected canonical pathways. On the other hand, comparison between the GR^iKO^ DSS and the GR^iKO^ control (Comparison 2) groups showed that LPS and IFNG were the upstream regulators activated, and IL10RA was inhibited, showing the greater change between the groups, while hepatic fibrosis, granulocyte adhesion and acute phase response signaling were the most commonly differentially regulated canonical pathways.

Moreover, comparison between the GR^flox^ DSS and GR^flox^ control groups (Comparison 3) showed that TGFB1 and lipopolysaccharide were the most differentially regulated upstream regulator molecules. IL10RA was, as predicted, inhibited, and hepatic fibrosis, and acute-phase response signaling were in the top differentially regulated canonical pathways, similar to Comparison 2. Interestingly, most of the top differentially regulated pathways and upstream regulators found in each comparison were inflammation-associated. When the number of differentially expressed genes was analyzed (Figure 3B), we found that the number for Comparison 2 was lower than for Comparisons 1 and 3, which had a similar number, although, the proportion of upregulated vs. downregulated genes was similar among all three groups (Figure 3C). Furthermore, we selected genes associated to the immune response and steroidogenic pathway that showed significant differences in any of the comparisons and grouped them in four different groups (steroidogenesis, cytokines, M1/M2 macrophage markers, and healing) to create a heatmap (Figure 3D). Comparisons 2 and 3 showed a greater number of upregulated genes in the cytokine, M1/M2 macrophage markers and re-epithelialization groups. *Ocln* was only downregulated in Comparison 2, while Comparison 1 showed fewer differentially expressed genes, such as upregulation of *St2* and *Il-6* (cytokines), *Cd40* (M1/M2 markers), *Ocln* (healing) and downregulation of *Tgfb1*. Interestingly, microarray analysis revealed that the *Nr5a2* transcript was downregulated in Comparison 2 (DSS-treated GR^iKO^ versus vehicle-treated GR^iKO^) (Figure 3E). Similarly, as observed in UC patients (Figure 2A), reduced *NR5A2* transcript levels were evident in the whole mucosa of the DSS-treated groups (Figure 3E), particularly in GR^iKO^ mice, and were related to extensive damage, compared to DSS-treated GR^flox^ mice (Table 2).

**Figure 4:**
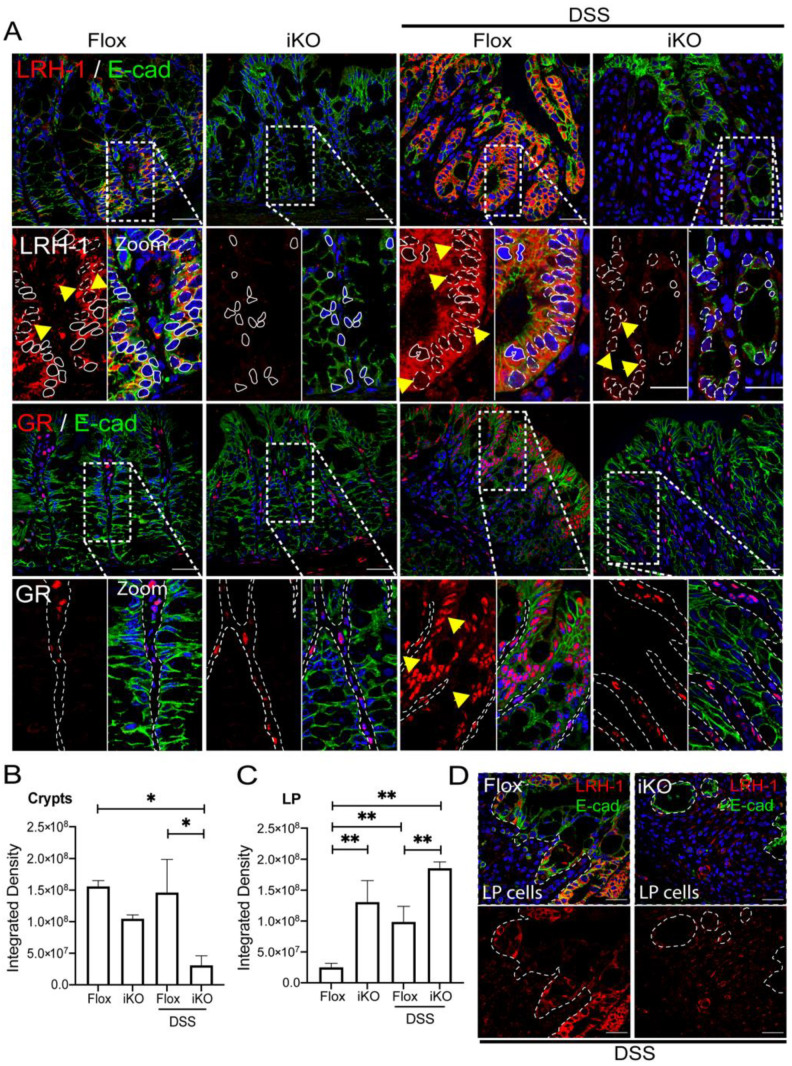
LRH-1 upregulated in intestinal mucosa lamina propria from DSS-treated GR^iKO^ mice. (**A**) Representative images of LRH-1 and total GR (red) immunofluorescent staining with co-localization of E-cadherin (green) as epithelial marker in intestinal mucosa from vehicle and DSS-treated GR^flox^ and GR^iKO^ mice. Hoechst was used for nuclear counterstaining. In the LRH-1 zoomed image: dashed line and/or yellow arrows: positive nuclear stain; solid line: negative nuclear stain. In the GR zoomed image, yellow arrows: positive nuclear stain; dashed line: epithelial outline. Objective 60×. Scale bar 30 μm. (vehicle *n* = 4 GR^flox^ and 3 GR^iKO^, DSS-treated *n* = 4 GR^flox^ and 6 GR^iKO^); (**B**) Integrated density from immunofluorescence images calculated from epithelial crypts; and (**C**) LP cells from intestinal mucosa of vehicle and DSS-treated GR^flox^ and GR^iKO^ mice, with (**D**) a representative image showing LRH-1 stain in LP from DSS-treated groups. Dashed line: epithelial outline. * *p* < 0.05, ** *p* < 0.01.

Additionally, LRH-1 stain was localized at the epithelium (Crypts) (Figure 4A,B) and LP cells (Figure 4C) in GR^flox^, but was reduced in GR^iKO^ mice in uninflamed conditions. Interestingly, a strong increase in cytosolic and nuclear LRH-1 staining was observed in the epithelium of DSS-treated GR^flox^ mice, with a slight immunoreactivity in DSS-treated GR^iKO^ mice (Figure 4A). In contrast, increased LRH-1 immunoreactivity, mostly localized in the cytoplasm and absent in the nuclei, was observed in the LP of the DSS-treated GR^iKO^ group compared to the vehicle-treated and GR^flox^ groups (Figure 4C, *p* ≤ 0.01). Moreover, total GR was higher in the nuclei of epithelial and LP cells in vehicle-treated GR^flox^ mice, contrasting with GR^iKO^ exclusively exhibiting GR immunoreactivity in LP cells (Figure 4A,C), as previously shown [33], confirming epithelium-specific intestinal GR deletion. Lastly, in DSS-treated GR^iKO^ mice, an increased nuclear GR distribution was mainly witnessed in LP, as opposed to the enhanced nuclear localization in the epithelial cells of DSS-treated GR^flox^ mice (Figure 4D).

The steroidogenic enzyme CYP11A1 was localized in most epithelial cell cytoplasm and present in both the vehicle-treated mice groups, with a higher content observed in the epithelial lining compared to epithelial crypt cells in GR^flox^ mice (Supplementary Figure S5). In GR^iKO^ mice, CYP11A1was distributed evenly across the crypt, with apical expression only preserved in DSS-treated GR^flox^ mice, decreasing in crypt epithelial cells in both GR^flox^ and GR^iKO^ mice.

### 3.4. GR-Dependent Activation of Steroidogenesis in Human Colonocytes

**Figure 5:**
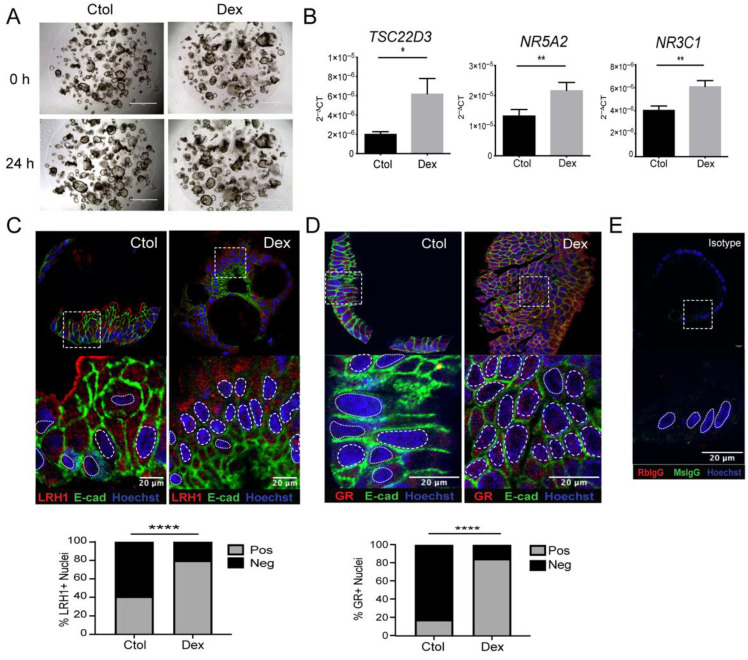
Dexamethasone induces LRH-1 in primary human intestinal organoids. Colonic organoids from healthy tissue were stimulated with 100 nM Dex for 24 h. (**A**) Organoids under light microscopy 4×. Scale bar 1000 μm. (**B**) Organoids were analyzed for *TSC22D3* (**left**), *NR5A2* (**center**) and *NR3C1* (**right**) transcript levels, relative to *18s* rRNA. Paired *t*-test, * *p* < 0.05; ** *p* < 0.01; *n* = 3. Representative immunoreactivity of (**C**) LRH-1 and (**D**) GR with corresponding (**E**) IgG isotype control in paraffin-embedded PFA-fixed sections of colonic organoids stimulated with 100 nM Dex for 24 h. Percentage of positive and negative nuclear staining for (**C**, **bottom**) LRH-1 and (**D**, **bottom**) GR. E-cadherin (E-cad, green) was used for epithelium counterstain and Hoescht as nuclear stain, **** *p* < 0.0001. In zoomed image: whole organoid for context purposes. Dashed line: positive nuclear stain; dotted line: negative nuclear stain. Objective 40×. Scale bar 20 μm.

To fully understand the effect of dexamethasone on the transcriptional regulation of LRH-1/*NR5A2*, we used human colonic organoids derived from non-inflamed non-IBD colonic tissue. The morphology of organoids was not evidently affected by 24 h dexamethasone (100 nM) treatment (Figure 5A). Dexamethasone treatment enhanced expression of the GC-response gene *TSC22D3* mRNA (encoding GILZ, a GC response target) in human colonic organoids (Figure 5B, *p* = 0.010). Dexamethasone significantly enhanced *NR5A2* mRNA levels (Figure 5B, *p* = 0.0016) and increased the percentage of LRH-1 positive-stained nuclei, as analyzed by immunofluorescence microscopy (Figure 5C). Similarly, dexamethasone treatment enhanced *NR3C1* mRNA levels (Figure 5B, *p* = 0.008) and increased GR-positive nuclei (Figure 5D). The isotype control showed non-immunoreactivity (Figure 5E).

**Figure 6:**
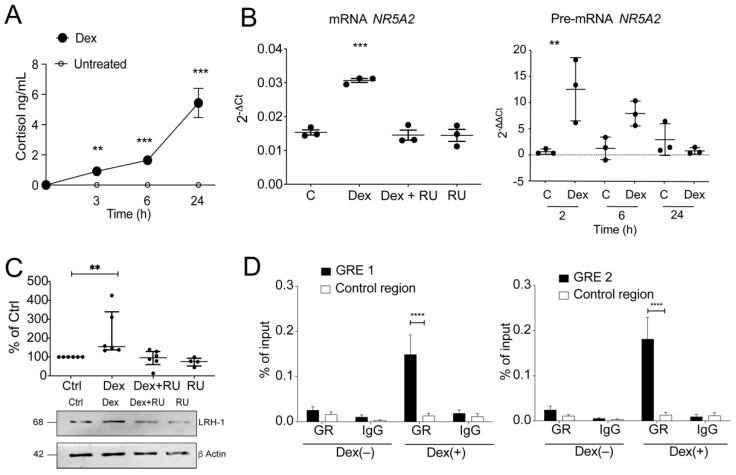
Dexamethasone induces cortisol and LRH-1, enhancing GR-binding to the *NR5A2* gene promoter in colonocytes. (**A**) The CCD841CoN colonocyte cell line was stimulated with dexamethasone (100 nM) for 3, 6 and 24 h, and cortisol levels were determined in supernatants by ELISA; (**B**) Cells upon Dex and/or GR antagonist RU-486 (10 µM) treatment for 8 h were used to evaluate mature (**left**) and precursor (**right**) *NR5A2* mRNA by RT-qPCR, and protein content by immunoblot; (**C**) Band quantification was performed by densitometry analysis and normalized to β-actin protein (% of control); (**D**) Cells stimulated with Dex for 2 h were analyzed for binding of GR to the *NR5A2* promoter by ChIP-qPCR; For each immunoprecipitated sample, the statistical analyses were performed with respect to an unrelated region from the *GAPDH* gene promoter. Results are expressed as % input ± SEM. One-way ANOVA with Tukey post-test was performed. ** *p* < 0.01, *** *p* < 0.001, **** *p* < 0.0001. C: control, Dex: dexamethasone, RU: RU-486, GRE: glucocorticoid responsive element, IgG: immunoglobulin G, nt: nucleotides; *n* = 4.

To examine the GR-mechanisms involved in LRH-1 expression and cortisol production in depth, we stimulated the CCD841CoN colonocyte cell line with Dex, observing a time-dependent increase in cortisol production at 3 (*p* < 0.01), 6 (*p* < 0.001), and 24 h. (*p* < 0.001) after 100 nM Dex stimulation (Figure 6A). Moreover, Dex also induced mature and precursor *NR5A2* transcript levels, an effect found to be GR-dependent, as mature mRNA increase was prevented by cell pre-treatment with RU-486 (Figure 6B). *NR5A2* transcript induction by Dex did not show significant differences between the concentrations used (Supplementary Figure S6A left). *NR5A2* induction was observed after 2 h of 100 nM Dex treatment, remaining at similar levels after 4 and 6 h (Supplementary Figure S6A right). LRH-1 protein upregulation was induced by Dex at 8 h (Figure 6C). Furthermore, *CYP11A1* mRNA levels did not vary with Dex concentration and time (Supplementary Figure S6B).

To validate these results and GR functionality, we analyzed *TSC22D3* induction by dexamethasone observing a concentration and time-dependent increase in gene expression, an effect counteracted by RU-486 (Supplementary Figure S6C), with no changes observed in *NR3C1* levels (Supplementary Figure S6D).

### 3.5. Glucocorticoids Regulate LRH-1 Expression by Binding to GREs in Regulatory Regions of the LRH-1/NR5A2 Gene

GCs induced LRH-1 expression within 2 h of treatment (Supplementary Figure S5A right), suggesting LRH-1 was a direct GR transcriptional target. In silico analysis of human *NR5A2* gene revealed the presence of two putative positive (+) GREs located in the regulatory region between 0.2 and 1.5 kb upstream of the transcription start site (each with a 74% score to the consensus GRE sequence according to the JASPAR database) [35].

Confirming direct LRH-1/*NR5A2* expression regulation by GR, the presence of GR binding to both GRE elements at the *NR5A2* promoter in dexamethasone-treated cells was detected using chromatin immunoprecipitation assays (ChIP) (*p* < 0.0001). The GR enrichment at each *NR5A2* GRE site was estimated using the GAPDH gene promoter as an internal control, with comparable GR levels to the negative control (IgG) in non-treated cells (Figure 6D).

To validate GR specificity in the *NR5A2* promoter binding, GR recruitment to the *TSC22D3* promoter GRE was confirmed in dexamethasone treated CCD841CoN cells (Supplementary Figure S7A, *p* < 0.05). Additionally, histone H3 enrichment at the *NR5A2* sites remained unaltered following dexamethasone treatment, comparable to that found in the *TSC22D3* promoter, suggesting absence of nucleosome chromatin remodeling at regulatory regions proximal to the transcriptional start site (Supplementary Figure S7B). These data demonstrate GR bound to the NR5A2 proximal promoter after dexamethasone treatment, and, therefore, induced LRH-1 expression in CCD841CoN cells.

## 4. Discussion

The principal therapeutic goal in UC is to achieve clinical and endoscopic remission, through drugs, such as azathioprine, biologicals, or small molecules, with corticosteroids currently being the first-line treatment in patients with moderate to severe UC [36]. Here, we demonstrated a novel GR-dependent activation and expression of the LRH-1 transcription factor, possibly by a positive feedback-loop, a process requiring participation of GREs located in the *NR5A2* promoter. Additionally, we found an association between LRH-1 expression and protein localization with steroid treatment response in UC patients’ mucosa and in a GRiKO colitis mice model, concluding that LRH-1 function involves GR regulation.

The intestinal mucosa from steroid-responding UC patients and control subjects produced cortisol in a GR- and LRH-1-dependent manner in ex vivo cultures, supporting the GR role in physiological intestinal steroidogenesis, as seen in adrenal gland cells [7]. However, Dex-induced cortisol production was lower in steroid-dependent or refractory patients than those responding to steroid treatment, being unaffected by LRH-1 blockade (SR-1848). This discrete response in cortisol production might be related to increased cytoplasmic LRH-1 localization in colonic epithelial and infiltrating cells of patients with altered GC response, reducing LRH-1 transcriptional activity. As reported, LRH-1 steroidogenic function required intact transcriptional activity, concomitantly reducing inflammatory scores and disease severity with the LRH-1 agonist 1,2-dilauroyl-sn-glycero-3-phosphocholine (DLPC) in a murine colitis model [37]. Conversely, inhibition of LRH-1 with SR-1848 in a hepatocyte cell line induced its translocation into the cytoplasm, decreasing transcriptional function and target gene expression [38]. Other factors influencing cytosolic LRH-1 permanency and activity comprise co-repressors and co-activators, such as the small heterodimer partner (SHP)/Dax-1 complex [39] and PGC-1a [40], respectively. Consequently, intestinal LRH-1 steroidogenic function was favored in a SHP−/− mice model of lymphocytic choriomeningitis virus infection [41] and was possibly affected when PGC-1a expression was decreased, as reported with respect to the inflamed mucosa of UC patients [42] and in a murine DSS colitis model [43]. Furthermore, cytosolic LRH-1 has been localized in colorectal cancer models [44], contributing to pathologic cell proliferation. However, due to the sample size in our UC-Rf and UC-D groups, further studies with a large multicentric patient cohort are needed to confirm an association between epithelial LRH-1 cytoplasmic distribution and cortisol production in UC.

Looking more deeply into GR participation in the mechanism underlying intestinal steroidogenesis, altered GR isoform content impacted Dex-induced cortisol production among patients. That UC-Rf or UC-D patients had higher GRβ transcript and nuclear protein content in epithelial cells might be partly explained by the lesser dexamethasone-induced cortisol levels and insensitivity to GR blockade in ex vivo culture supernatants. Similarly, GR expression and cellular localization has been related to steroid therapy response in UC patients [4,45,46], with inflammatory signaling pathways increasing the GRβ/GRα ratio in epithelial and lymphoid-derived cell lines [47].

The importance of GR in intestinal homeostasis, and particularly the steroidogenesis process, was demonstrated in the GR^iKO^ DSS-treated mice which exhibited, as expected, diminished LRH-1 expression in crypt cells. Additionally, LRH-1 content was increased in LP associating with inflammatory features, thus, recapitulating LRH-1 expression in patients with altered GC response.

We observed the induction of the *NR5A2* transcript and LRH-1 protein in Dex-stimulated human colonic organoids. However, the *NR5A2* transcript content results were somewhat unexpected in the patient samples; it is well documented, that in many other genes, transcript and protein levels do not necessarily correlate [48,49]. Therefore, our results could be explained by the effect of the UC inflammatory environment reducing LRH-1 protein degradation or turnover [50,51,52]. Moreover, we observed increased LRH-1 in immune lamina propria cells, and, as others have shown, LRH-1 was critical in T-cell function and activation and in a T-cell adoptive transfer colitis murine model. Therefore, we conclude that LRH-1 is important in epithelial and immune cells involved in UC pathophysiology (see graphical abstract). In this context, particularly when GR signaling is capped by uncontrolled inflammation mediated by NF-κB and GRβ variant expression, LRH-1-mediated steroidogenesis is attenuated. Further studies of LRH-1 post-translational modifications should be undertaken for a deeper understanding of this phenomenon.

## 5. Conclusions

In conclusion, steroid-dependent and steroid-refractory patients showed similar alterations in the steroidogenic pathway, both having elevated GRβ accompanied by high cytoplasmic LRH-1 content, possibly explaining impaired cortisol production. The interplay between LRH-1 and GR demonstrates their mutual participation in maintaining intestinal homeostasis. Prospectively, the LRH-1/GRβ profile, revealed as beneficial for the differentiation of steroid responder patients from those becoming dependent/refractory after steroid therapy, offers the chance to switch to a more suitable therapy before relapse/failure.

## Figures and Tables

**Table 1:** Clinical and demographic characteristics of enrolled subjects.

Demographic/Clinical Data	Healthy Controls *n* = 10	Ulcerative Colitis Patients		
		Responders *n* = 13	Steroid-Refractory *n* = 4	Steroid-Dependent *n* = 4
Gender (*n*; %) Male Female	5 (50) 5 (50)	6 (46) 7 (54)	2 (50) 2 (50)	1 (25) 3 (75)
Age in years (median; range)	53 (41–66)	32 (20–63)	29 (22–38)	38 (24–41)
Body mass index (median; range)	26 (20.7–29.8)	24.1 (16.9–29.8)	22 (14.7–32.1)	21 (18–26.3)
Smoking habit (*n*; %)	1 (10)	1 (8)	1 (25)	1 (25)
Family history of IBD (*n*; %)	0 (0)	2 (15)	0 (0)	1 (25)
Years of disease (median; range)	-	1 (0–8)	1 (1–2)	8 (3–20)
Extraintestinal manifestations (*n*; %)	-	5 (38)	0 (0)	3 (75)
Montreal classification (*n*; %) E1: Extensive colitis E2: Left-sided colitis E3: Proctitis	-	11 (85) 2 (15) 0 (0)	3 (75) 1 (25) 0 (0)	3 (75) 1 (25) 0 (0)
IBD current treatment (*n*; %) 5—Aminosalicylates Azathioprine Methotrexate 6-Mercaptopurine	-	9 (69) 2 (15) 1 (8) 1 (8)	4 (100) 0 (0) 0 (0) 0 (0)	2 (50) 2 (50) 0 (0) 0 (0)
Clinical Mayo score (median; range)	-	4 (1–8)	4 (2–7)	5 (3–5)
Endoscopic Mayo score 2 (Moderate activity) 3 (Severe activity)	-	9 (69) 4 (31)	2 (50) 2 (50)	3 (75) 1 (25)
Fecal calprotectin (median; range)	-	939 (254–2410)	600 (258–1320)	640 (600–1200)

**Table 2:** Clinical and histopathological scores from DSS mice models.

Evaluation	GRflox (*n* = 10)	GRiKO (*n* = 8)
Rectal bleeding (% of mice at day 7)	40	59
Rectal bleeding severity (% of mice at day 7)	5% severe, 20% mild	18% severe, 36% mild
Colon length (cm)	6.7 ± 0.2	5.9 ± 0.1
Body weight loss (% of initial weight) ***	91 ± 1	86 ± 1
Disease activity index (DAI) ***	1.8 ± 0.2	3.15 ± 0.15
Pathological Scores:		
Inflammation (*p* = 0.06)	2.3 ± 0.4	3.4 ± 0.3
Erosion *	2.1 ± 0.6	3.8 ± 0.1
Atrophy	2.9 ± 0.5	3.7 ± 0.2
Fibrosis	2.5 ± 0.4	2.9 ± 0.1
Edema *	1.3 ± 0.5	3 ± 0.7

* *p* < 0.05; *** *p* < 0.001.

## Data Availability

The Gene Expression Omnibus accession number for the microarray data set is GSE146048 (https://www.ncbi.nlm.nih.gov/geo/query/acc.cgi?acc=GSE146048). Last accessed on 20 December 2021.

## Supplementary Materials

The following supporting information can be downloaded at: https://www.mdpi.com/article/10.3390/cells11121905/s1, Figure S1. Evaluation of Th1, Th2 and Th17 cytokines in colonic mucosa of healthy and ulcerative colitis patients, Figure S2. *CYP11A1* levels in intestinal mucosa from UC patients and controls, Figure S3. LRH-1 and GRβ are upregulated in lamina propria from mucosa of UC patients with impaired GC response, Figure S4. Representative images of hematoxylin-eosin-stained mucosa from vehicle- and DSS- treated GR^Flox^ and GR^iKO^ mice, Figure S5. CYP11A1 expression in epithelia of DSS-GR^iKO^ mice, Figure S6. GR-dependent LRH-1 gene induction in colonocytes, Figure S7. Dexamethasone promotes GR binding to the *TSC22D3* (GILZ) gene promoter. Reference [53] are refered to in Supplementary Materials.
