# Supplementary material for: Regulation of the Intestinal Extra-Adrenal Steroidogenic Pathway Component LRH-1 by Glucocorticoids in Ulcerative Colitis
Source: Cells. 2022 Jun 12;11(12):1905. doi: 10.3390/cells11121905 (PMC9221003; doi:10.3390/cells11121905)
Supplement: Supplementary file 1 [file cells-11-01905-s001.zip › cells-1677520-supplementary.pdf]

cells

MDPI

Supplementary Materials

Regulation of the Intestinal Extra-Adrenal Steroidogenic Pathway Component LRH-1 by Glucocorticoids in Ulcerative Colitis

Glauben Landskron [1,2,†], Karen Dubois-Camacho [1,†], Octavio Orellana-Serradell [1,†], Marjorie De la Fuente [1,2], Daniela Parada-Venegas [1,3], Mirit Bitrán [1], David Diaz-Jimenez [4], Shuang Tang [5], John A. Cidlowski [4], Xiaoling Li [6], Hector Molina [1], Carlos M. Gonzalez [7], Daniela Simian [8], Jaime Lubascher [9], Victor Pola [10,11], Martín Montecino [10,11], Tjasso Blokzijl [3], Klaas Nico Faber [3], María-Julieta González [12], Rodrigo Quera [13] and Marcela A. Hermoso [1,*,‡]

[1] Innate Immunity Laboratory, Immunology Program, Biomedical Sciences Institute, Faculty of Medicine, Universidad de Chile, Santiago 8380453, Chile; glandskron@uft.cl (G.L.); kdubois@med.uchile.cl (K.D.-C.); octavio.orellana.s@gmail.com (O.O.-S.); mdelafuente@uft.cl (M.D.l.F.); dparadav@gmail.com (D.P.-V.); mirit.bitran@ug.uchile.cl (M.B.); hectormol@gmail.com (H.M.)
[2] Biomedicine Research Laboratory, Medical School, Universidad Finis Terrae, Santiago 7501015, Chile
[3] Department of Gastroenterology and Hepatology, University Medical Center Groningen, University of Groningen, 9713 GZ Groningen, The Netherlands; t.blokzijl@umcg.nl (T.B.); k.n.faber@umcg.nl (K.N.F.)
[4] Molecular Endocrinology Group, Signal Transduction Laboratory, National Institute of Environmental Health Sciences, National Institutes of Health, Department of Health and Human Services, Research Triangle Park, Durham, NC 27709, USA; david.diaz-jimenez@nih.gov (D.D.-J.); cidlows1@niehs.nih.gov (J.A.C.)
[5] Metabolism and Nuclear Medicine Group, Fudan University Cancer Institute, Fudan University Shanghai Cancer Center, Shanghai 200433, China; tangshuang@fudan.edu.cn
[6] Metabolism, Genes, and Environment Group, Signal Transduction Laboratory, National Institute of Environmental Health Sciences, National Institutes of Health, Department of Health and Human Services, Research Triangle Park, Durham, NC 27709, USA; lix3@niehs.nih.gov
[7] School of Veterinary Medicine, Faculty of Life Sciences, Universidad Andrés Bello, Santiago 8370251, Chile; carlosgonzalez@unab.cl
[8] Academic Research Unit, Clínica Las Condes, Santiago 7591018, Chile; danielasimian@gmail.com
[9] Inflammatory Bowel Disease Program, Gastroenterology Department, Clínica Las Condes, Santiago 7591018, Chile; jlubascher@clinicalascondes.cl
[10] Institute of Biomedical Sciences, Faculty of Medicine and Faculty of Life Sciences, Universidad Andrés Bello, Santiago 8370186, Chile; vpola02@ug.uchile.cl (V.P.); mmontecino@unab.cl (M.M.)
[11] FONDAP Center for Genome Regulation, Santiago 8370146, Chile
[12] Cell and Molecular Biology Program, Biomedical Sciences Institute, Faculty of Medicine, Universidad de Chile, Santiago 8380453, Chile; jgonzale@med.uchile.cl
[13] Centro de Enfermedades Digestivas, Programa Enfermedad Inflamatoria Intestinal, Clínica Universidad de Los Andes, Universidad de los Andes, Santiago 7620157, Chile; rquera@clinicauandes.cl
* Correspondence: mhermoso@med.uchile.cl; Tel.: +56-2-29786572; Fax: +56-2-29786979
† These authors contributed equally.
‡ Current Address: Innate Immunity Laboratory, Immunology Program, Biomedical Sciences Institute, Faculty of Medicine, Universidad de Chile, Independencia 1027, Independencia, Santiago 8380453, Chile.

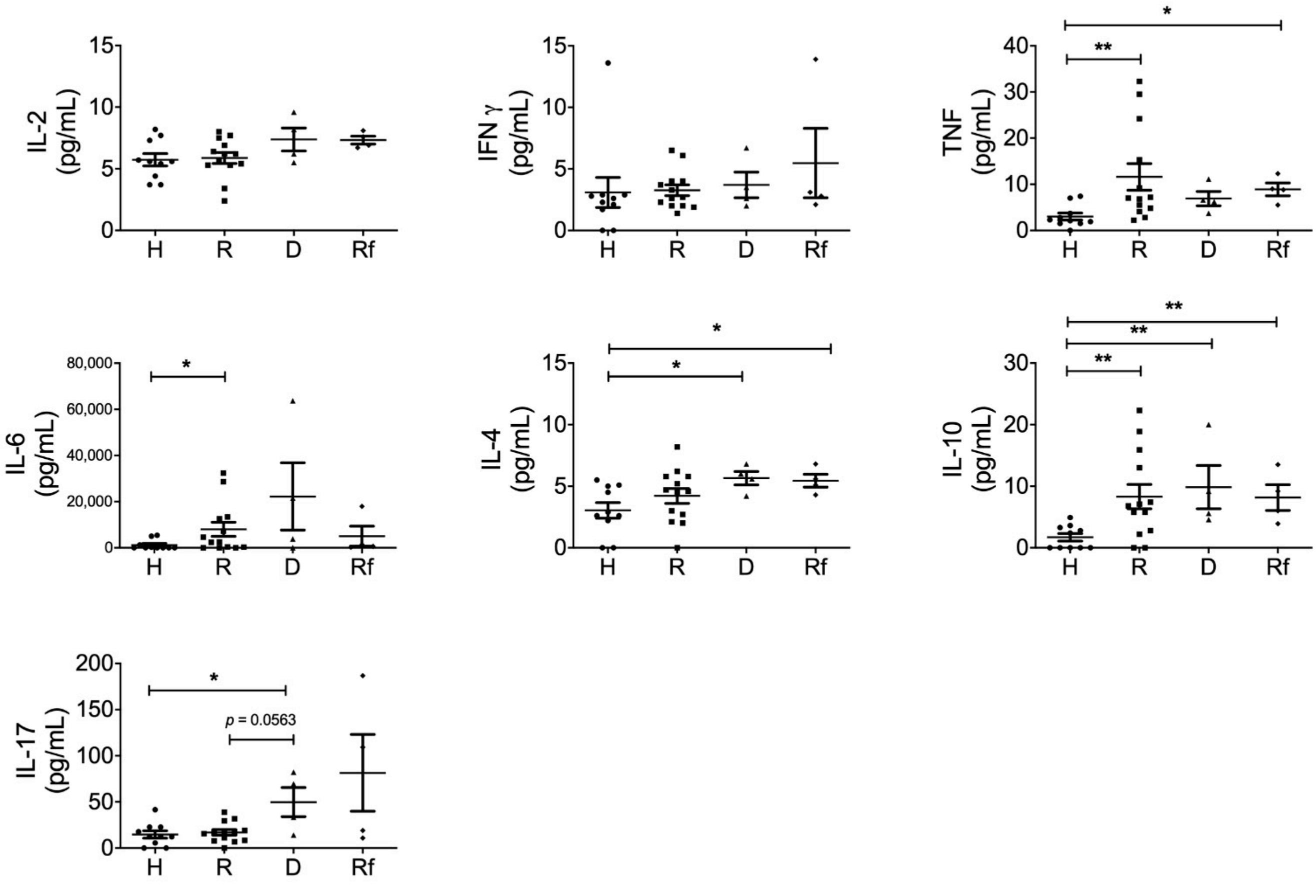

Figure S1. Evaluation of Th1, Th2 and Th17 cytokines in colonic mucosa of healthy and ulcerative colitis patients. Supernatants of ex vivo biopsies from healthy controls (H, circles), responders (R, squares), dependent (D, triangles) and refractory (Rf, diamonds) patients were analyzed for cytokine production by CBA assay. $*p < 0.05$, $** p < 0.001$. Differences between group medians were assessed using Kruskal–Wallis with Dunn's post-test, $* p < 0.05$; $** p < 0.01$. Each point represents an individual subject.

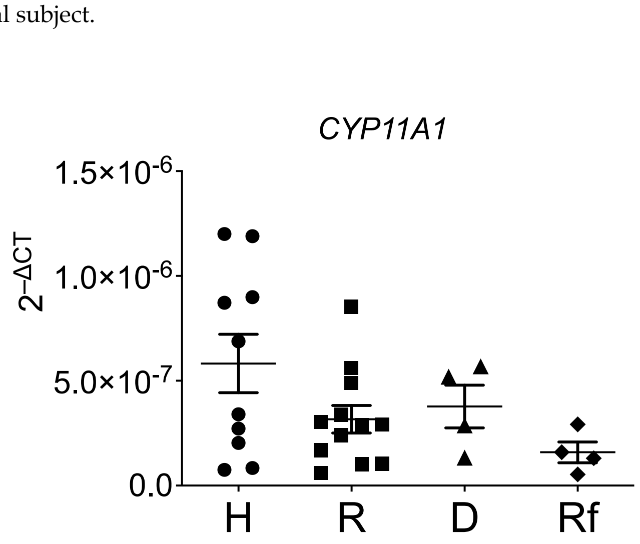

Figure S2. *CYP11A1* levels in intestinal mucosa from UC patients and controls. The expression of steroidogenic enzyme *CYP11A1* mRNA (relative to *18s* rRNA) from healthy controls (H, circles), responders (R, squares), dependent (D, triangles) and refractory (Rf, diamonds) patients was determined by RTqPCR. Differences between medians were assessed using Kruskal–Wallis with Dunn's post-test . Each point represents an individual subject.

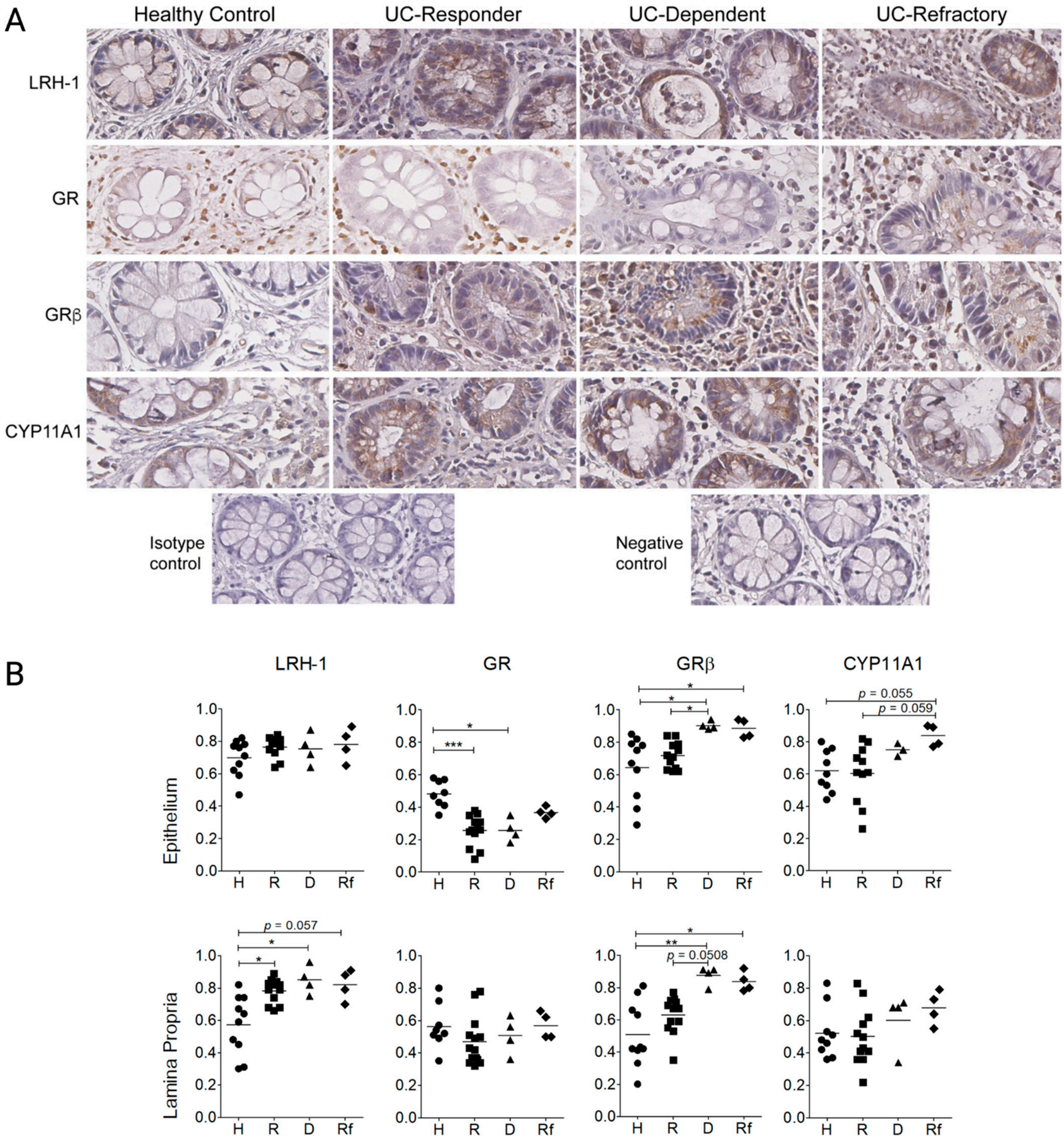

Figure S3. LRH-1 and GRβ are upregulated in lamina propria from mucosa of UC patients with impaired GC response. (**A**) Representative immunoreactivity from intestinal mucosa samples of healthy controls (H, circles), responders (R, squares), dependent (D, triangles) and refractory (Rf, diamonds) patients for LRH-1, GR, GRβ and CYP11A1 were determined in paraffin-embedded PFA-fixed sections; objective 40X. Negative control: reagent diluent without primary antibody; (**B**) Positivity index from immunostaining in epithelium and lamina propria of LRH-1, GR and GRβ and CYP11A1. Differences between medians were assessed using Kruskal–Wallis with Dunn's post-test. * $p < 0.05$; ** $p < 0.01$, *** $p < 0.001$. Each point represents an individual patient: healthy (circles), responders (squares), dependents (triangles) and refractory (diamonds).

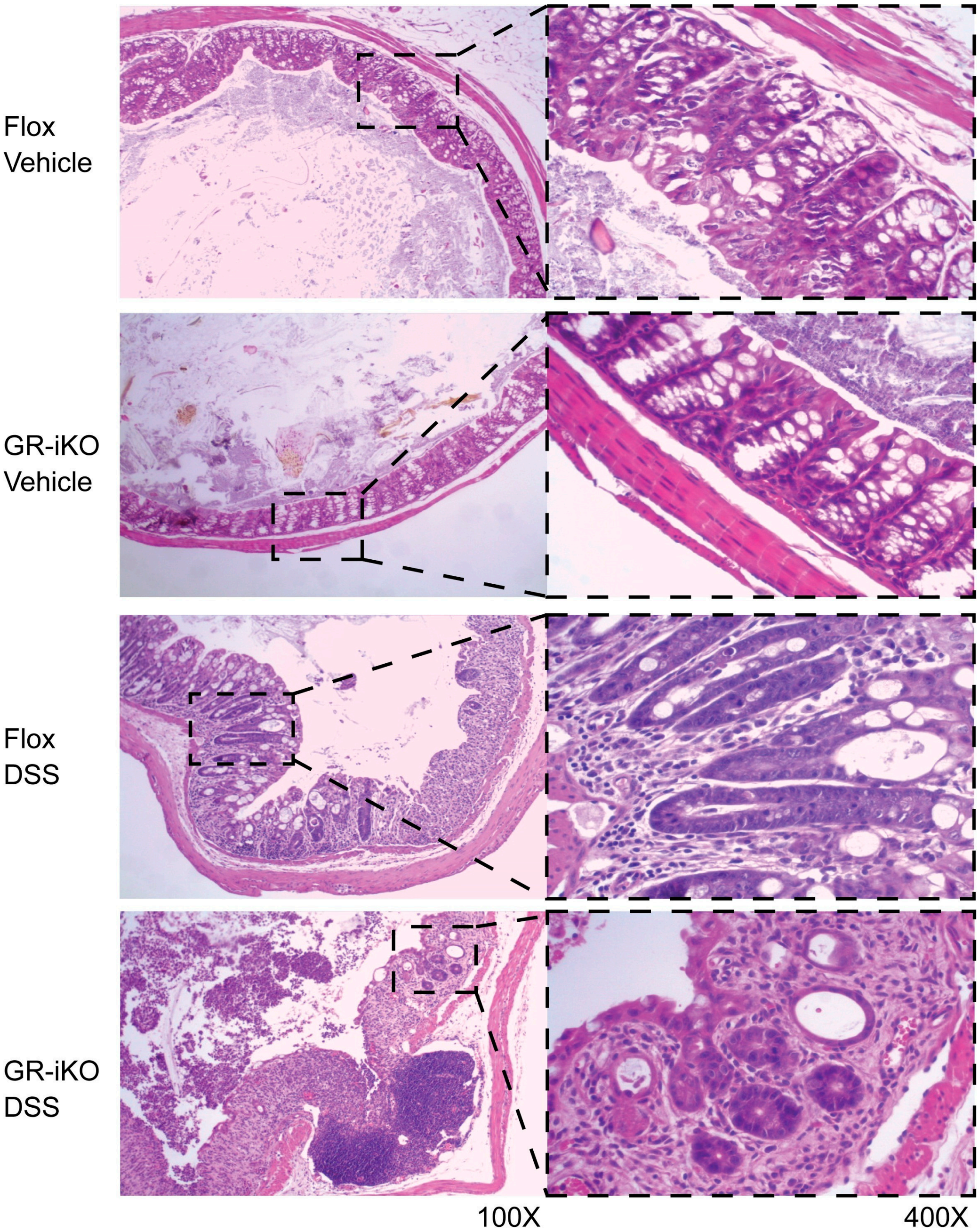

Figure S4. Representative images of hematoxylin-eosin-stained mucosa from vehicle- and DSS-treated GR^{Flox} and GR^{iKO} mice. Images were taken at 100X (left column) and 400X (right column) from dashed lined squares.

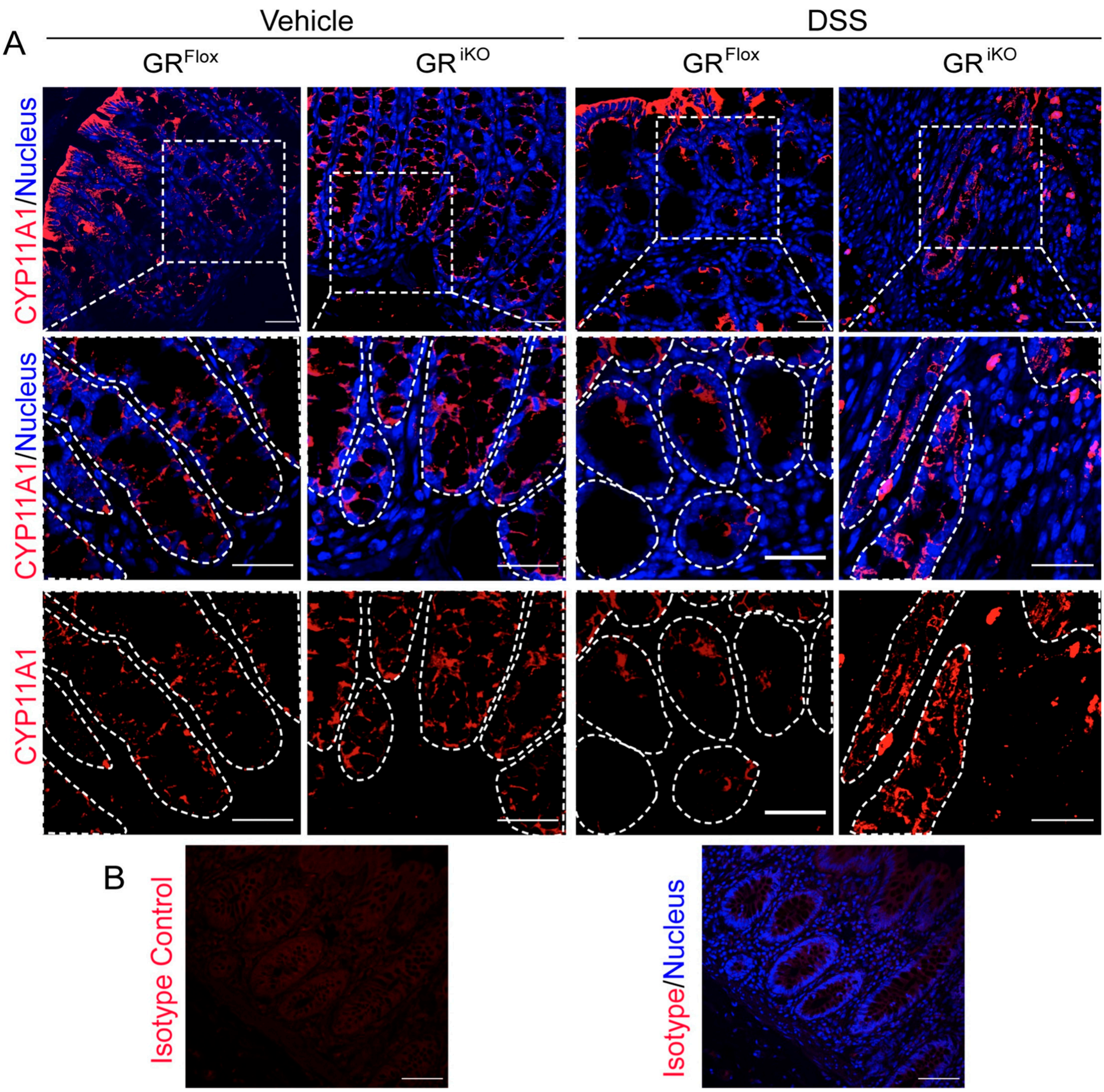

Figure S5. CYP11A1 expression in epithelia of DSS-GR^{iKO} mice. (**A**) Representative immunoreactivity from intestinal mucosa samples of GR^{Flox} and GR^{iKO} mice under vehicle and 2.5% DSS treatment were determined in paraffin-embedded PFA-fixed sections; objective 20X. Scale bar: 30um. (**B**) Isotype control in normal human intestinal mucosa; objective 20X. Scale bar: 30um.

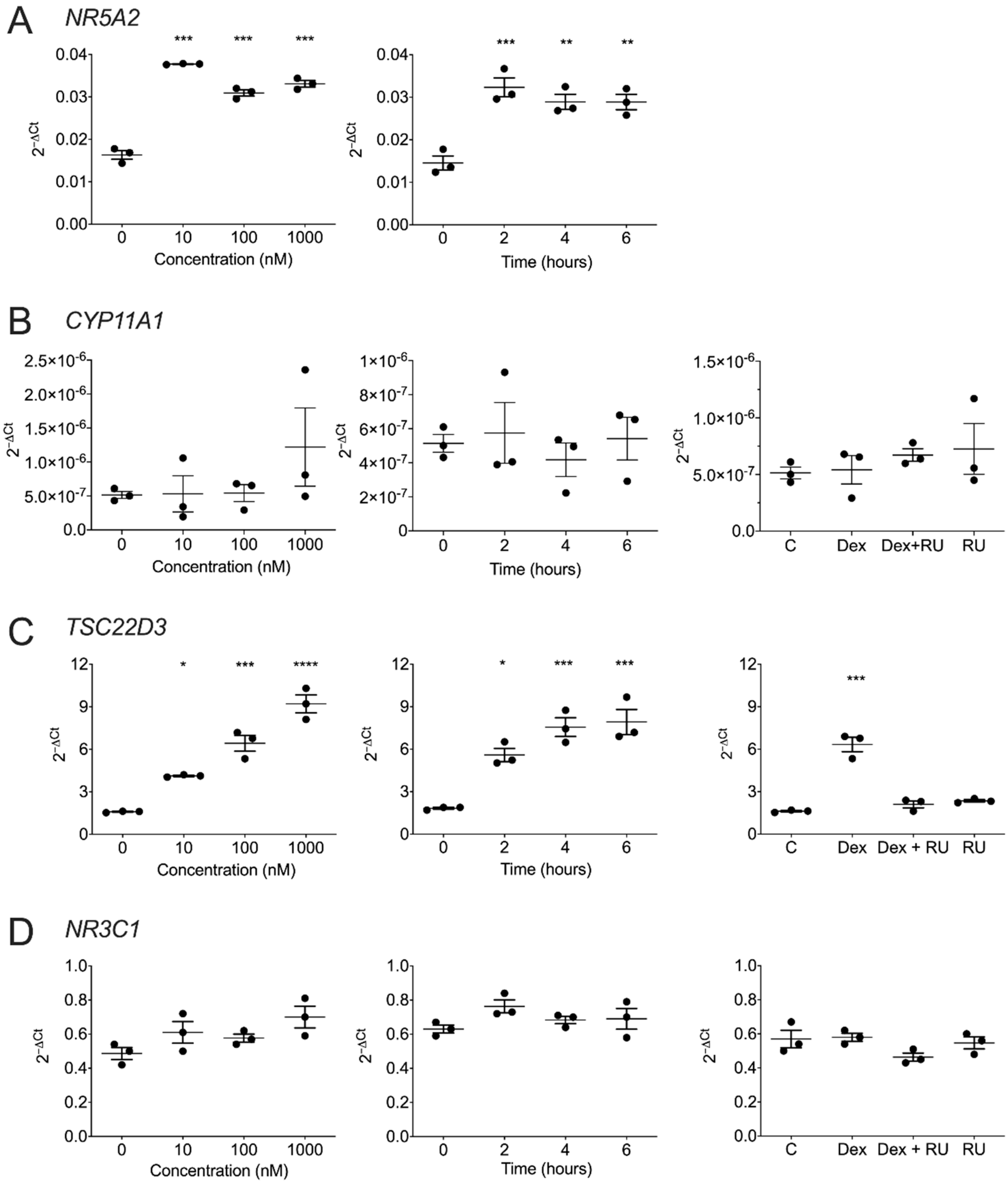

Figure S6. GR-dependent LRH-1 gene induction in colonocytes. CCD841CoN cells exposed to different doses of dexamethasone for 6 h were analyzed for gene expression (left panel), time-dependent expression with a single dose of 100nM at 2, 4 and 6 hrs (middle panel) and in response to dexamethasone (100 mM) and/or GR antagonist RU-486 (10 μM) (right panel). Cell lysates were analyzed for (**A**) *NR5A2*, (**B**) *CYP11A1*, (**C**) *TSC22D3*, and (**D**) *NR3C1* transcript levels by RT-qPCR. One-way ANOVA with Bonferroni post-test, * $p < 0.05$, ** $p < 0.01$, *** $p < 0.001$, **** $p < 0.0001$. $n = 3$.

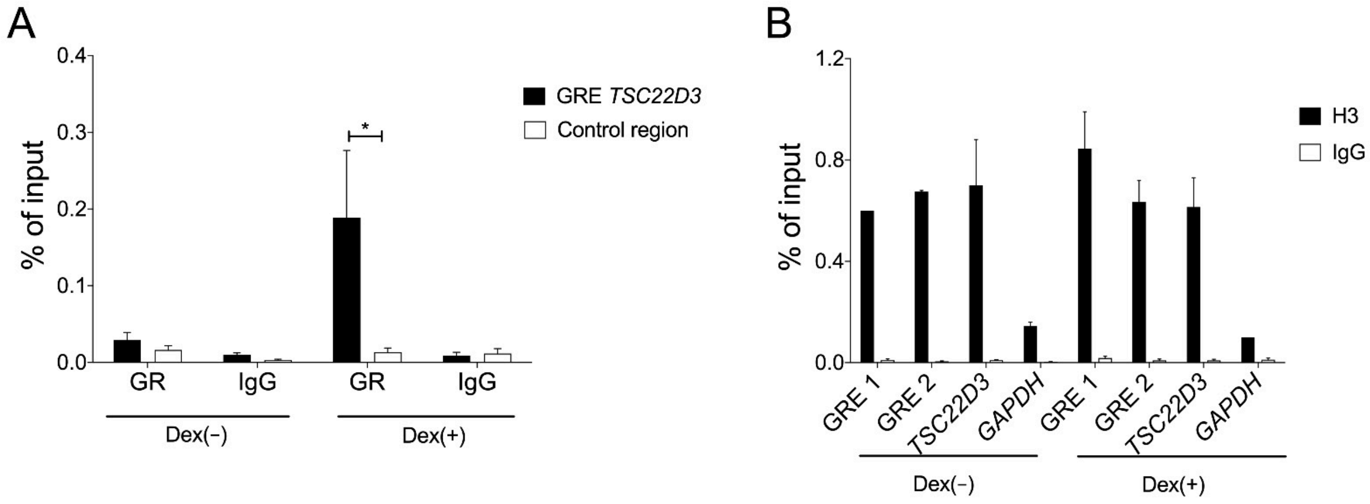

Figure S7. Dexamethasone promotes GR binding to the *TSC22D3* (GILZ) gene promoter. To control for positive GR binding, our precipitated chromatin samples were also analyzed for the previously reported GR association with the (**A**) *TSC22D3* gene promoter in dexamethasone-stimulated (100 nM, 2 hr.) CCD841CoN cells. Statistical analyses were performed with respect to the unrelated *GAPDH* gene promoter region, as shown in Figure 4C; (**B**) Histone 3 enrichment values were used to demonstrate significant and comparable chromatin precipitations for the *NR5A2*-GRE1, *NR5A2*-GRE2, *TSC22D3*-GRE and *GAPDH* sequences. Results were expressed as % of the input samples. The corresponding normal IgG values indicate the specificity during each chromatin immunoprecipitation analysis. One-way ANOVA with Tukey post-test was performed, * $p = < 0.05$. GRE: glucocorticoid responsive element, IgG: immunoglobulin G, H3.3A: histone 3. $n = 4$.

Supplementary methodology

Dex and RU-486, were purchased from Steraloids, Inc (Newport, RI, USA). LRH-1 inhibitor, SR-1848, was purchased from Sigma-Aldrich (Saint Louis, MO, USA. Cat. SML1513). Culture media: MEM (Corning®, Manassas, VA, USA), DMEM/F12 (Carlsberg, CA, USA). Bovine serum (FBS) and hormone-depleted charcoal-stripped FBS (Hyclone, Carlsbad, CA, USA). Antibiotics: gentamicin (Sigma Aldrich, Saint Louis, MO, USA); fungizone (Gibco, ThermoFisher Scientific, Waltham, MA, USA); normocin (Invivogen, San Diego, CA, USA); penicillin/streptomycin P/S (100 U/ml penicillin + 100 μg/ml streptomycin, Gibco, Thermo Scientific, Waltham, MA, USA).

Organoid culture reagents: Cultrex™ UltiMatrix BME (R&D systems, Minneapolis, MN, USA); R-Spondin-1 (R&D Systems, Abingdon, UK); Noggin (R&D Systems, Minneapolis, MN, USA); 1X B27 (Gibco, ThermoFisher Scientific, Waltham, MA, USA); N-acetylcysteine (Sigma-Aldrich, Saint Louis, MO, USA); EGF (Gibco, ThermoFisher Scientific, Waltham, MA, USA); IGF-1 (Gibco, ThermoFisher Scientific, Waltham, MA, USA); FGF-2 (Gibco, ThermoFisher Scientific, Waltham, MA, USA); nicotinamide (Sigma-Aldrich, Saint Louis, MO, USA); SB202190 (Sigma-Aldrich, Saint Louis, MO, USA); Y-27632 (Sigma-Aldrich, Saint Louis, MO, USA); A83 (Tocris Bioscience, Minneapolis, MN, US). Wnt-conditioned medium was produced using L-Wnt-3a cells (ATCC, Manassas, VA, USA).

Antibodies: Polyclonal rabbit anti-LRH-1 antibodies (Novus Biologicals, Centennial, CO, USA Cat. NBP2-27196 for western blot (1:1000) and indirect immunofluorescence (1:100); Atlas Antibodies, Bromma, Sweden, Cat. HPA017067 for immunohistochemistry); polyclonal rabbit anti-GR (1:200, Cell Signaling Technologies, Beverly, MA, USA, Cat. 12041); polyclonal rabbit anti-GRβ isoform (1:1000, Thermo-Fisher, Waltham, Massachusetts, Cat. PA3-514); polyclonal rabbit anti-CYP11A1 (1:200, Cell Signaling Technologies, Danvers, MA, Cat. 14217S); CYP11B1 (1:50, Biorbyt, Cambridge, UK, Cat. Orb5937); monoclonal mouse anti-E-cadherin (1:200, BD Biosciences, Franklin Lakes, NJ, USA, Cat.

610181); and monoclonal mouse anti-actin antibodies (1:1000, Santa Cruz Biotechnology, Dallas, TX, USA Cat. 47778). Secondary antibodies diluted in 1:200 donkey anti-mouse Alexa Fluor 488-conjugated (Cat. A-21202), and donkey anti-rabbit Alexa Fluor 647-conjugated (Cat. A-31573) were used and purchased from Invitrogen (Thermo-Fisher, Waltham, Massachusetts). HRP-conjugated secondary antibodies goat anti-rabbit and goat anti-mouse (1:10000 dilution each) for western blot were purchased from Invitrogen (Catalogs # G-21234 and # 31432, respectively, Thermo-Fisher, Waltham, Massachusetts). Monoclonal rabbit anti-H3 (histone 3) for ChIP was purchased from Abcam (Abcam Cambridge, MA, USA, cat. ab176842)

TaqMan® Assays-on-demand primer (Thermo Fisher Scientific, Thermo-Fisher, Waltham, Massachusetts, USA): *NR5A2*/LRH-1 (Hs00187067_m1); mouse *Nr5a2*/Lrh-1 (Mm00446088_m1); *NR3C1*/GR (Hs00353740_m1, Exons 4-5); *NR3C1*/GR-β (Hs00354508_m1, Exons 8-9) [53], *TSC22D3*/GILZ (Hs00608272_m1); *PPIB* (Hs00168719_m1); mouse Rn18s (Mm03928990_g1). Primers 5′-3′ for SYBR Green qPCR: rRNA 18s (Fwd GTGGAGCGATTTGTCTGGTT; Rev CGCTGAGCCAGTCAGTGTAG), *CYP11A1,* NM_000781.2:425-580 (Fwd TGGCTGCATGGGACGTGATTTT; Rev TCCTCGAAGGACATCTTGCTGTCT), *CYP11B1,* NM_000497.3; NM_001026213.1 (located in exon junction 2-3 Fwd TTGCTGAATGGGCCTGAAT; located in exon junction 3-4 Rev GCCAAGTTGCTGGCTTCTAT). The sequence for qPCR primers and probes for ChIP analysis were: *NR5A2*-GRE1 (Fwd CCTGCATAGAGTCATGTGATGAG; Rev CCAACATTAGTGACCTGCTGTA; Probe ACCATCATGAAACTGGATACATGGT), *NR5A2*-GRE 2 (Fwd CGGGTAGATCATTTGAGGTCAC; Rev CCAGTAGCTGGGATTACAGG; Probe AAATTAGCCGGGCATGGTGGTGT), *TSC22D3*-GRE (Fwd TTCAATGGGTACTGGCCTTAAC; Rev GGTGATGCAACCGGGAATA; Probe TGTGGTGGAACCCAATGTTCTCCT). *GAPDH* (Fwd CGGCTACTAGCGGTTTTACG; Rev AAGAAGATGCGGCTGACTGT).

q-PCR

Total RNA from each sample was extracted with the RNEasy Mini kit (Qiagen, Hilden, Germany) following the manufacturer’s protocol, and integrity was analyzed by electrophoresis in 1% agarose gel and amounts determined by spectrophotometric analysis in a Synergy 2 microplate reader (Biotek Instruments Inc., Winooski, VT, USA). Two µg of RNA was used for DNAse assay according to the manufacturer’s instructions (Ambion, Carlsbad CA USA). Then, one µg of RNA was used to synthesize cDNA using oligo-dT (Thermo Fisher Scientific, Waltham, MA, USA) with RT-AffinityScript enzyme (Agilent Technologies Inc., Santa Clara, CA, USA) at a final volume of 20 µL.

Immunohistochemistry (IHQ)

Paraformaldehyde-fixed mucosa samples from UC patients and healthy controls were organized into a single paraffin cassette and corresponding slides were dewaxed and rehydrated. Then, slides were incubated for 10 min at 95°C–100°C in antigen retrieval buffer (pH 6.0). After cooling, slides were blocked with PBS (2% BSA). The sections were incubated overnight at 4°C with the corresponding primary antibodies for LRH-1, total GR, GRβ and CYP11A1. Positive and negative controls were run with each batch of patient/study slide tested. For negative controls, we included an IgG isotype control in parallel to specific antibody staining. Endogenous peroxidase was inhibited with 3% H_2O_2 for 10 min and then a universal secondary antibody (VECTASTAIN® Universal Quick HRP Kit (Peroxidase), R.T.U, Vector Laboratories, Burlingame, CA, USA, Cat. PK-7800) was added to the sections for 30 min at 37°C. Slides were revealed with DAB+ Substrate Chromogen kit (Dako, Carpinteria, CA, USA) followed by counterstaining with Harris hematoxylin. Coverslips were mounted with Entellan® medium (Merck, Darmstadt, Germany). Positive and negative controls were run with each batch of patient/study slides tested. Immunostaining analysis is included in the Supplementary Material. For

immunostaining analysis, images were captured with Aperio ScanScope, and analyzed with the Aperio ImageScope Software (Version 10.3.2) (Leica Biosystems, Deer Park, IL, USA). The analysis was performed selecting epithelium and lamina propria separately with pen tools in the whole biopsy, and positive pixels for LRH-1, CYP11A1, GR and GRβ. were evaluated with the algorithm Positive Pixel Count 9. This analysis included all pixels in each cell of the colon biopsies; the proportion of positive pixels relative to total pixels per area (positive and negative) was considered as the positivity index, then validated by two experienced pathologists.

Cortisol measurement

Briefly, 50 μL of standards or samples were added in duplicate to plate wells, 75 μl of assay buffer were added to the non-specific binding wells and 50 μL of assay buffer were added to the maximum binding wells. Then, 25 μL of the DetectX cortisol conjugate and 25 μL of the DetectX cortisol antibody were added to each well (except the NSB wells) and the plate was placed in a shaker for 1 h at room temperature. Later, the plate was washed and dried by tapping it on absorbent towels and 100 μL of the TMB Substrate was added to each well and incubated for 30 min at room temperature. The reaction was concluded by adding 50 μL of the stop solution to each well. The optical density of cortisol from each well was read at 450 nm in a Biotek Synergy 2 plate reader (Biotek Instruments Inc., Winooski, VT, USA) within 15 min after adding the stop solution. Cortisol concentrations for each well were calculated according to the manufacturer's instructions using a 4PL regression model with standard curve.

Chromatin Immunoprecipitation (ChIP) assays

Briefly, cells were precipitated by centrifugation, lysed using a cell lysis buffer containing protease inhibitors (cOmplete™ Protease Inhibitor Cocktail, Sigma-Aldrich, Saint Louis, MO, USA) and mechanic homogenization, and then nuclear lysis was performed using a nuclear lysis buffer. Chromatin fragmentation was performed by sonication (10 cycles of 30 seconds (sec), each followed by a 30 sec pause) (Bioruptor UCD-200, Diagenode, Sparta, NJ, USA). After 10 cycles, samples were incubated on ice for 10 min, followed by another 10 cycles of sonication and finally repeated once more, completing 30 cycles in total. The amount and fragmentation of chromatin was evaluated by incubating 30 μL of each sample with RNAse A (Merck Millipore, Burlington, MA, USA) for 30 min at 37°C, Proteinase K (Merck Millipore, Burlington, MA, USA) for 120 min at 62°C and then running it in a 1% agarose gel. Once fragmentation was confirmed, chromatin immunoprecipitation was carried out by incubating DNA fragments sized between 0.2 and 0.5 kb using 3 μg of anti-GR monoclonal antibody overnight, mixed with 20 μL of protein A/G PLUS-Agarose (Santa Cruz Biotechnology, Dallas, TX, USA). Chromatin was then separated from protein A/G using successive washes with a low salt, high salt, LiCl and TE buffer, followed by reversion of the protein/chromatin union by incubating with proteinase K for 2 h at 62°C and then 10 min at 95°C. Afterwards samples were cooled at room temperature and the DNA was purified using phenol/chloroform extraction. Finally, the obtained DNA was used for qRT-PCR, as described above, to amplify the selected glucocorticoid response elements (GREs). Each sample Ct value was analyzed in triplicate, comparing the initial chromatin input (by using a dilution curve), expressed as a percentage of each condition enrichment vs. *GAPDH* gene enrichment, and used as the internal control. Additionally, a GRE located in the *TSC22D3* promoter (a glucocorticoid-response gene) was used as a positive control for Dex-induced gene expression.

Western blot

Cell lysates were immersed in Laemmli Buffer (Sigma Aldrich, Saint Louis, MO, USA), boiled at 95°C x 5 min, and 15 μg were loaded in 10% polyacrylamide gels. Protein transference and chemiluminescence are detailed in the supplementary methods.

Protein transference to nitrocellulose membranes was accomplished using the Trans-Blot Turbo Transfer System (Bio-Rad Laboratories Inc. Hercules, CA, USA). Membranes were blocked with Odyssey®-PBS blocking buffer (LI-COR, Lincoln, NE, USA) for 1 h at room temperature, then incubated overnight at 4°C with the corresponding primary antibody, followed by incubation with the appropriate secondary antibody for 1 h at room temperature. Chemiluminescence was evaluated using ChemiScope equipment (ClinX Science Instruments, Shanghai, China), and bands were quantified with ImageJ Software (Version 1.53q) (US National Institutes of Health, Bethesda, ML, USA).
